# Neural-specific deletion of mitochondrial p32/C1qbp leads to leukoencephalopathy due to undifferentiated oligodendrocyte and axon degeneration

**DOI:** 10.1038/s41598-017-15414-5

**Published:** 2017-11-09

**Authors:** Mikako Yagi, Takeshi Uchiumi, Noriaki Sagata, Daiki Setoyama, Rie Amamoto, Yuichi Matsushima, Dongchon Kang

**Affiliations:** 1 Department of Clinical Chemistry and Laboratory Medicine, Graduate School of Medical Sciences, Kyushu University, 3-1-1, Maidashi, Higashi-ku, Fukuoka 812-8582 Japan; 2 Department of Nutritional Sciences, Faculty of Health and Welfare, Seinan Jo Gakuin University, 1-3-5 Ibori, Kokurakita-ku, Kitakyushu 803-0835 Japan

## Abstract

Mitochondrial dysfunction is a critical step in the pathogenesis of many neurodegenerative diseases. The p32/ C1qbp gene functions as an essential RNA and protein chaperone in mitochondrial translation, and is indispensable for embryonic development. However, little is known about the consequences of mitochondrial dysfunction of p32 deletion in the brain development. Here, we found that mice lacking p32 in the central nervous system (p32cKO mice) showed white matter degeneration accompanied by progressive oligodendrocyte loss, axon degeneration and vacuolation in the mid brain and brain stem regions. Furthermore, p32cKO mice died within 8 weeks of birth. We also found that p32-deficient oligodendrocytes and neurons showed reduced oligodendrocyte differentiation and axon degeneration in primary culture. We show that mitochondrial disruption activates an adaptive program known as the integrated stress response (ISR). Mitochondrial respiratory chain function in oligodendrocytes and neurons is, therefore, essential for myelination and axon maintenance, respectively, suggesting that mitochondrial respiratory chain dysfunction in the central nervous system contributes to leukoencephalopathy.

## Introduction

Mitochondria are responsible for the generation of ATP through oxidative phosphorylation (OXPHOS) and also play vital roles in β oxidation and Ca^2+^ buffering, and in the production of pro-apoptotic factors and reactive oxygen species (ROS)^1,2^. It has been suggested that mitochondrial failure plays a role in a wide variety of diseases that involve tissues with high-energy demand, such as neurodegenerative diseases^3–5^. Leukoencephalopathy with brainstem and spinal cord involvement is a progressive disorder characterized by abnormalities in the white matter of the central nervous system that results from mitochondrial dysfunction^6^.

p32 [also termed Complement C1q Binding Protein (C1QBP)] is conserved among eukaryotic organisms and is primarily localized in the mitochondrial matrix, being associated with several matrix proteins^7,8^. Previously, we found that p32-deficient mice exhibited mid-gestation lethality and p32-deficient mouse embryonic fibroblast (MEF) cells showed severe dysfunction of the mitochondrial respiratory chain because of severely impaired mitochondrial protein synthesis. p32-deficient MEF cells also showed reduced OXPHOS function. The binding of p32 to mitochondrial RNA and mitochondrial ribosomes correlates with mitochondrial translation, suggesting that p32 is an RNA and protein chaperone required for functional mitochondrial ribosome formation^9^. p32 also regulates metabolism to maintain the respiratory chain complex and oxidative phosphorylation in cancer cells^10^ and is highly expressed in prostate cancer^11^. A mutation of p32 has been suggested as a cause of mitochondrial respiratory chain disorder^12^.

Oligodendrocytes are responsible for myelination of axons in the central nervous system. At birth, axons are initially unmyelinated and become myelinated during development. Oligodendrocyte precursor cells grow into immature oligodendrocytes, and finally into mature, myelin-producing oligodendrocytes, a process that largely occurs between P10 and P60^13,14^. Demyelination occurs in multiple mitochondrial diseases, including Leber’s hereditary optic neuropathy^15^, dominant optic atrophy^16^ and mitochondrial encephalomyopathy, lactic acidosis and stroke-like episodes (MELAS)^17^. These reports suggest that mitochondrial functions are required for proper oligodendrocyte differentiation and myelination^18^.

In the early postnatal development of neurons, aerobic metabolism is the dominant metabolic pathway when lipids and proteins are needed for the processes of axonal elongation, synaptogenesis, and myelination. On the other hand, oxidative phosphorylation in neurons is important for synaptic activity throughout development and adulthood^19,20^. Although encephalopathy is characterized by deep gray matter involvement as one of its more prevalent clinical features, white matter involvement has been increasingly recognized as a common feature in patients with mitochondrial diseases^21^.

To explore a potential role of p32 in neurodegeneration *in vivo*, we generated and analyzed neural-specific conditional p32 knockout (p32cKO) mice using the nestin-Cre-loxP approach. We indicate that mitochondrial respiratory chain dysfunction in oligodendrocytes causes oligodendrocyte undifferentiation, resulting in myelin destruction, which leads to myelin ballooning and vacuolation. We also found that mitochondrial respiratory chain dysfunction in neurons led to axon degeneration with increased cleaved caspase 3 activity, indicating that mitochondrial respiratory chain function is also involved in axonal maintenance.

## Results

### Mice lacking p32 in the nervous system show leukoencephalopathy

p32-deficient mice exhibit mid-gestation lethality associated with a severe developmental defect^9^. To identify the potential role of p32 in the central nervous system, floxed p32 (p32^loxP/loxP^) mice, in which exon 3 of the p32 gene is flanked by two loxP sequences, were crossed with nestin-Cre transgenic mice. Nestin-Cre mice are well established to induce specific recombination of floxed genes, mainly in neurons and glial cells (oligodendrocytes and astrocytes) starting at E13.5–E14.5^22^. In contrast to the embryonic lethality of the complete p32 knockout mouse, p32^flox/flox^; nestin-Cre (p32cKO) mice were born at the expected Mendelian ratio.

**Figure 1:**
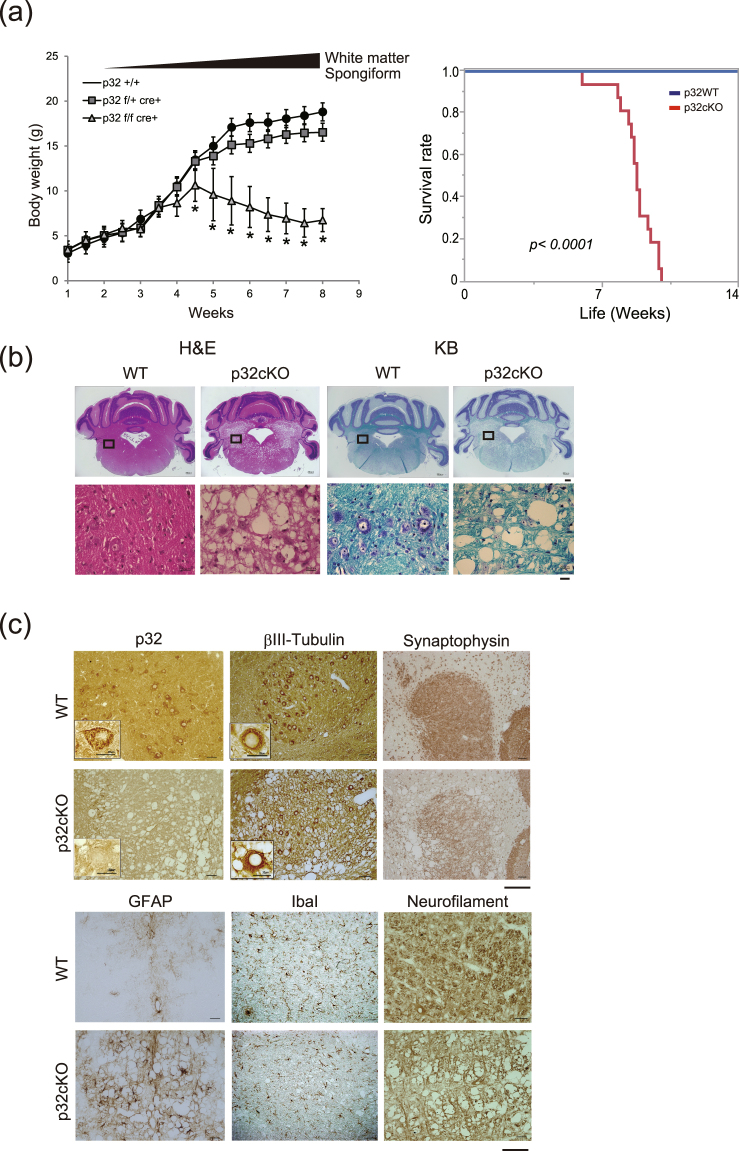
Mice lacking p32 in the nervous system show weight loss and progressive spongiosis. (**a**) Body weight curves of wild-type (p32+/+), heterozygous (p32+/flox:nestin-cre), and p32 knockout (p32flox/flox:nestin-cre) mice. KO mice weigh significantly less than wild-type and heterozygous mice; n = 4, depending on age and genotype. *p < 0.05 for all time points beyond postnatal day 28 (P28) as assessed by two-way ANOVA. (Right Panel) Survival curves for p32f/f (WT) and p32f/f nestin-cre (p32cKO) mice. (n = 16 for each genotype). *p < 0.0001 versus corresponding group. (**b**) Histological analysis of sagittal sections of the cerebellum from 6-week-old control and p32cKO mice. Coronal cerebellar sections were stained with hematoxylin & eosin (HE) and Kluver-Barrera (KB) stains. Lower panels show high magnification view of respective areas. Bars = 200 μm (upper) and 20 μm (lower). (**c**) Sections of 6-week-old wild-type and p32cKO cerebellum were analyzed by immunohistochemistry with anti-p32, βIII-tubulin, GFAP, Synaptophysin, IbaI and Neurofilament. Scale bars = 50 μm. Cerebellar nuclei cells are shown in the p32 and βIII-tubulin panels. Scale bar = 20 μm.

The mutant mice showed growth retardation compared with control littermates. At P21, the p32cKO mice began to lose weight, they showed tremor and all mutant mice were dead by 8 weeks (Fig. 1a). The size of the p32cKO brain was almost the same as that of control mice and no anatomical abnormalities were detected in the p32cKO brain (Supplementary Fig. 1a). To evaluate deletion of p32 in the mutant mice, lysates were prepared from various regions of the brain. p32 was efficiently deleted in several regions of the mutant brain, including the cerebral cortex, midbrain, medulla, and cerebellum (Supplementary Fig. 1b).

### p32 deficiency results in progressive vacuolization and spongiosis in white matter

To study neuronal defects in p32cKO mice, histological analyses were performed on the brains of control and mutant mice at 6 weeks after birth. Brain development and neural migration were examined by H&E staining and were observed to be intact and the cell bodies of the neurons appeared normal in p32cKO mice (Fig. 1b). Brain sections from p32cKO mice showed moderate bilateral and symmetrical vacuolation (spongiform degeneration) within the pons, medulla and midbrain, but not in the cerebral or cerebellar cortices (Fig. 1b). These small vacuoles were first observed in the pons at 2 weeks of age and, thereafter, vacuoles number and size were greatly increased compared with control mice (Supplementary Fig. 2a). These vacuoles were negative for lipids by staining with Oil red O (data not shown). p32cKO brain sections stained with Kluver–Barrera (KB) showed severe demyelination (myelin breakdown) or hypo-myelination throughout the white matter (Fig. 1b). Similar but smaller vacuoles were also present in the spinal cord (Supplementary Fig. 2b). Taken together, these results suggest that deletion of p32 by nestin-Cre leads to reduced development of white matter.

### p32 deficiency results in astrogliosis and reduced synaptogenesis

The brain is made up of many cells, including neurons and glial cells. To investigate neuron and glial cell morphology, we used several marker antibodies. p32 was clearly expressed in the wild-type nervous system; however, no obvious expression was observed in the p32cKO brain (Fig. 1c). The neuronal cell marker βIII-tubulin was expressed and localized in p32cKO somata and well-developed neurites, similar to wild-type mice, suggesting that neural cell bodies were intact in p32-deficient neurons. A significant decrease in synaptophysin immunoreactivity found in the cerebellar nucleus of p32cKO mice suggested synaptic loss. Neurofilament immunohistochemistry showed many small bundles of disrupted axons throughout the white matter in p32cKO mice, suggesting that axon degeneration occurred in white matter regions (Fig. 1c).

Astrogliosis is among the most common features of neurodegeneration. GFAP immunoreactivity was markedly increased in the vacuolated areas of the white matter, especially in the pons, medulla and midbrain regions (Fig. 1c). Together, these results suggest that degeneration of axons and dendrites is likely to be responsible for the loss of normal neuronal functions in p32cKO mice. Microglia can have both positive and detrimental roles in inflammatory processes. We found that Iba1- or CD11b-positive cell numbers were not increased in 6-week-old p32cKO mice, suggesting no inflammation (Fig. 1c and Supplementary Fig. 2c).

### Oligodendrocyte degeneration

**Figure 2:**
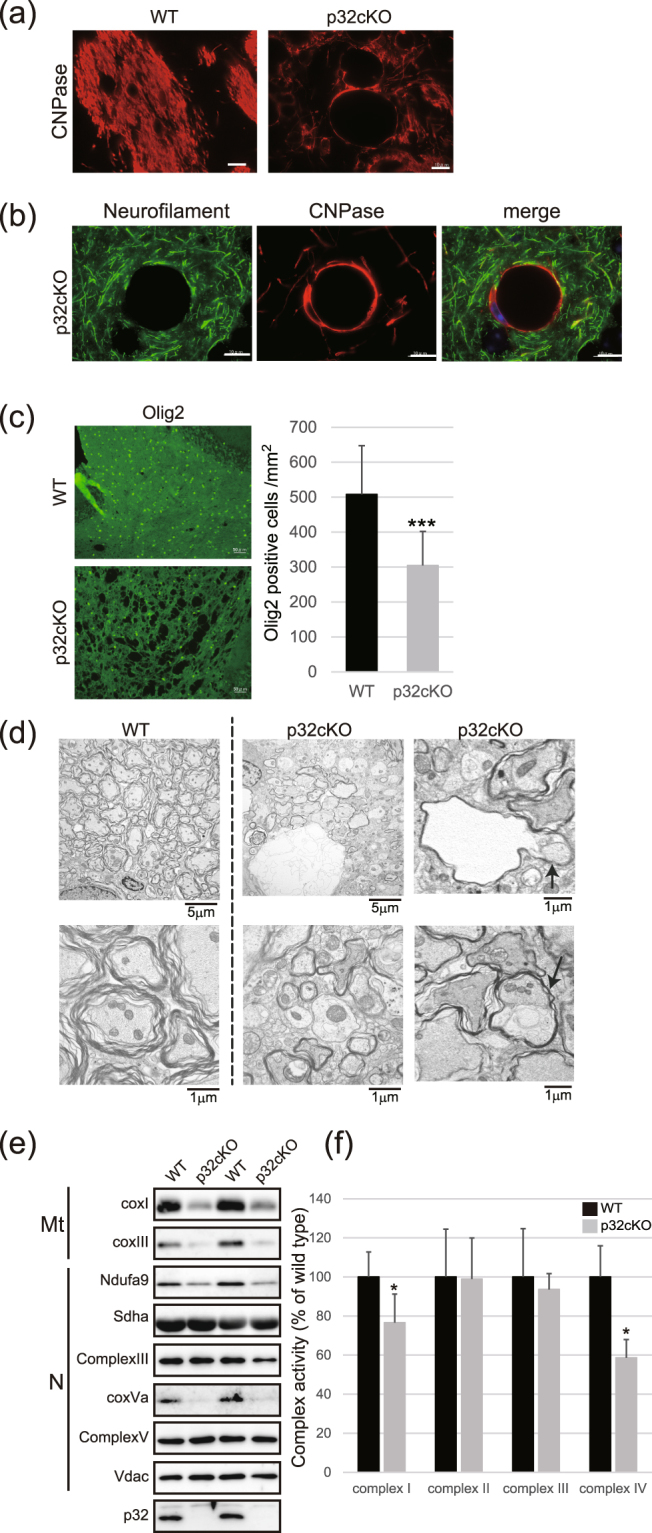
Loss of p32 causes oligodendrocyte and neurite degeneration. (**a**) Cerebellar sections from 6-week-old WT and p32cKO mice were analyzed by immunohistochemistry for the oligodendrocyte marker, 2′,3′-Cyclic-nucleotide 3′-phosphodiesterase (CNPase). Scale bars = 10 μm. (**b**) Cerebellar sections from 6-week-old p32cKO mice were analyzed by double-immunohistochemistry for CNPase [for oligodendrocytes (red)] and Neurofilament [for neuronal axons (green)]. Scale bars = 10 μm. (**c**) Loss of Olig2-positive nuclei correlated with a notable decrease in oligodendrocyte lineage cells within the white matter of the cerebellar nuclei in p32cKO mice compared with controls. ***p < 0.001 vs. control. Scale bars = 50 μm. (**d**) Transverse sections of the pons at 5 weeks of age in wild-type (left panel) and p32cKO mice showing thinly myelinated or non-myelinated axons of the white matter in cerebellar nuclei. Arrow shows axons that have degenerated as a result of compression from vacuoles within their myelin sheaths. Abnormal and swelled mitochondria with compact cristae are present in p32cKO brains. Scale bars = 5 μm and 1 μm. (**e**) Expression of mitochondrial proteins. Brain extracts were prepared from equal amounts of brain white matter regions (N = 6, each group). Mt: mitochondrial-encoded OXPHOS protein; N: nuclear-encoded OXPHOS protein. (**f**) Submitochondrial particles (SMPs) derived from p32cKO brains had reduced mitochondrial complex I and complex IV activity. Assays were performed in triplicate. ^∗^p < 0.05 by Mann–Whitney test.

Oligodendrocytes are essential for myelination and metabolic support of motor axons. Cerebellar sections from p32cKO and control mice were analyzed by immunohistochemistry using antibodies against 2′,3′-cyclic nucleotide 3′-phosphodiesterase (CNPase) and myelin basic protein (MBP), a structural component of myelin. Similar to H&E staining of the white matter, we detected significant oligodendrocyte pathology of spongiosis, demyelination and hypomyelination (Fig. 2a and Supplementary Fig. 2d). Interestingly, the vacuoles appeared to be embedded in the circle of CNPase staining, suggesting that these vacuoles may be generated by degeneration of oligodendrocytes (Fig. 2b). Furthermore, a small number of Olig2-staining cells (a marker for oligodendrocyte precursor cells) observed in the demyelinated areas of p32cKO mice was attributed to either a myelination or a remyelination failure because of the absence of oligodendrocyte precursor cells (Fig. 2c). These data suggest that abnormal oligodendrocytes and hypomyelination were caused by a myelination deficiency in the mutant mice.

### Hypo-myelination and myelin ballooning in p32cKO mice

Further examination of white matter sections by transmission electron microscopy showed the presence of apparent hypo-myelination with aberrant myelin sheaths, and myelin ballooning (vacuoles associated with the myelin sheath) in the p32 mutant mice (Fig. 2d). These periaxonal space vacuoles, with or without associated axons, were increased in size and number compared with the wild type, and vacuole spaces were seen from the outermost surface of the myelin sheath. Axons sometimes appeared to have degenerated because of compression from vacuoles within their myelin sheaths. Moreover, electron microscopic examination of p32cKO nerves revealed collapsed or swollen mitochondria in neurons (Fig. 2d), thus confirming that nestin-specific deletion of p32 resulted in mice with disrupted mitochondrial morphology in central neurons and oligodendrocytes.

### Reduced mitochondrial translation and complex activity

Previously, we observed that p32 is involved in mitochondrial translation and that complexes I, III and IV are strongly reduced in p32 KO MEF cells^9^. Therefore, we sought to investigate possible roles for p32 in mitochondrial translation and OXPHOS activity in brain tissue. There was no decrease in mitochondrial DNA copy number or mtRNA expression in the p32cKO brain compared with control mice (Supplementary Fig. 3). We observed severe depletion of the mtDNA encoded proteins, mt-CoxI, CoxIII and of nuclear encoded Ndufa9 and CoxVα in p32cKO nerves compared with controls (n = 6, each group) (Fig. 2e). We also found that the activities of complexes I and IV were significantly reduced in p32cKO brain mitochondria, while the activities of complex II and III were unchanged (Fig. 2f). These findings suggest that p32 in the brain might also be involved in mitochondrial translation and mitochondrial respiratory chain activity.

### Oligodendrocyte differentiation and axon degeneration

**Figure 3:**
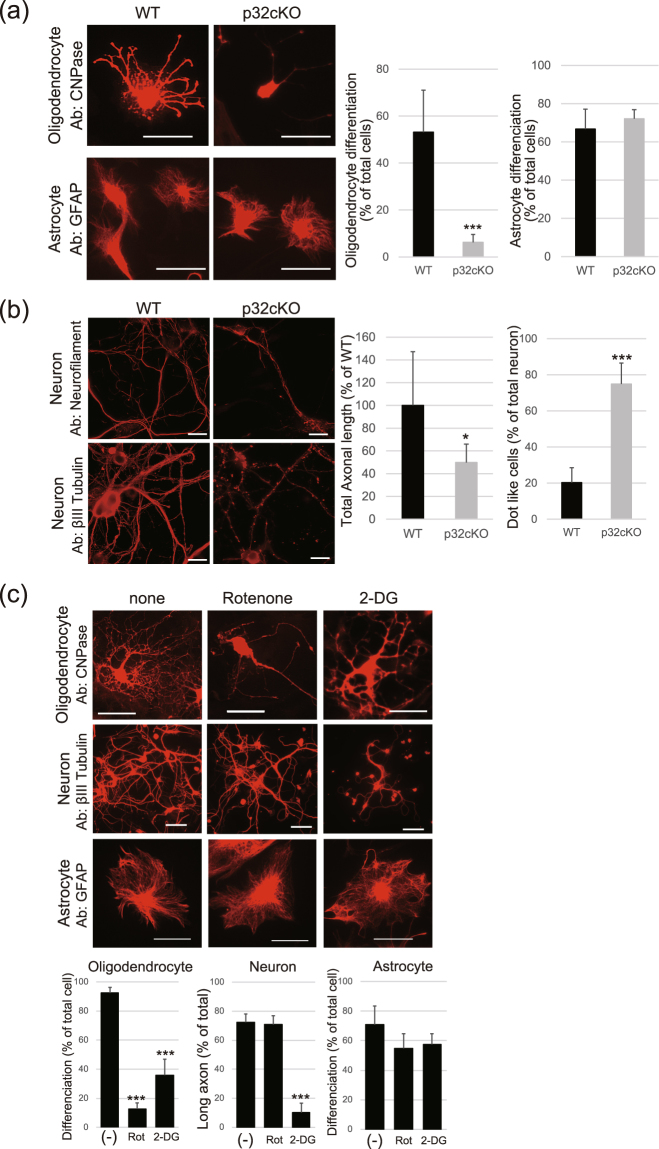
Reduced oligodendrocyte differentiation and axon degeneration. (**a**) Characterization of oligodendrocyte differentiation and astrocyte growth in 1 week-old cortical cultures. After culture for 7 days, cells were reacted with the indicated antibody, CNPase for oligodendrocytes and GFAP for astrocytes. Quantitation of well differentiated oligodendrocytes and astrocytes demonstrated a significant reduction of oligodendrocyte differentiation in p32cKO. Scale bars = 50 μm. (**b**) Length of axons immunostained with neurofilament antibody was decreased in p32cKO. Dot-like structures in neurons identified by βIII-tubulin staining were increased in p32cKO neurons after culture for 2 weeks. Scale bars = 50 μm. (**c**) Sensitivity of oligodendrocyte differentiation and neurite outgrowth to rotenone (1 nM) or 2-deoxy glucose (2-DG)(10 mM). Isolated oligodendrocytes, neurons and astrocytes were treated with the complex I inhibitor, rotenone, or 2-DG. After culture for 7 days, cells were stained with each marker (CNPase, βIII-tubulin and GFAP) and the ratio of oligodendrocyte differentiation, neurite outgrowth and astrocyte differentiation was measured. Scale bar = 50 μm.

Oligodendrocyte precursor cells grow into immature, and finally into mature oligodendrocytes 10 days after birth^14^. Because hypomyelination and myelin ballooning in p32cKO mice and wild-type myelination occur after 2 weeks, we hypothesized that differentiation of oligodendrocytes was affected in p32cKO mice. First, we investigated oligodendrocyte differentiation in primary culture. In p32-deficient oligodendrocyte precursors, oligodendrocyte differentiation was reduced compared with that in the wild type, suggesting that p32 function in oligodendrocytes might be required for proper oligodendrocyte differentiation (Fig. 3a). In astrocyte cultures stained for GFAP there was no difference between wild-type and p32-deficient astrocytes, suggesting that p32 is not involved in astrocyte growth *in vitro* (Fig. 3a and Supplementary Fig. 4).

To examine the role of p32 in axon and dendrite elongation in central neurons, we prepared primary cortical neuron cultures for both mutant and wild-type mice. Immunostaining with βIII-tubulin showed that neurite growth was similar between KO and wild-type primary neurons for 7 days of growth; however, there were some βIII-tubulin dot structures at 13 days in the p32-deficient primary neurons (Fig. 3b). We also found that immunostaining analysis of neurofilaments showed that axon elongation was decreased in p32cKO neurons (Fig. 3b). This sequence of events occurs similarly during neuritic degeneration. We confirmed p32 protein deletion in these primary cell cultures (Supplementary Fig. 4). These results suggest that p32 in neurons is involved in axon maintenance but not neurite elongation.

### Rotenone inhibits oligodendrocyte differentiation and axon maintenance

Next we investigated whether mitochondrial respiratory chain function or glycolysis affected neurite growth or maintenance and oligodendrocyte differentiation. We first tested the effects of mitochondrial respiratory chain inhibition during the oligodendrocyte differentiation process by treating cells with 1 nM rotenone, which inhibits complex I, or with 10 mM 2-DG, which inhibits glycolysis. We found that rotenone completely inhibited and 2-DG partially inhibited oligodendrocyte differentiation, suggesting that oligodendrocyte differentiation depends substantially on mitochondrial respiratory chain activity and partially depends on glycolysis (Fig. 3c and Supplementary Fig. 5). 2-DG, however, completely inhibited neurite outgrowth, suggesting that neurite outgrowth depends on the glycolysis process (Fig. 3c and Supplementary Fig. 5). In contrast, we found that rotenone did not inhibit neurite outgrowth, suggesting that mitochondrial respiratory chain function was not essential for neurite outgrowth. We also observed that 2-DG, but not rotenone, completely inhibited neurite outgrowth of p32cKO neurons, suggesting that neurite outgrowth depends on the glycolysis process, even in respiratory chain deficient neurons (p32cKO) (Supplementary Fig. 6).

We showed that rotenone and 2-DG did not inhibit astrocyte outgrowth, suggesting that respiratory chain function and glycolysis were less involved in astrocyte growth compared with that in neurons and oligodendrocytes (Fig. 3c).

**Figure 4:**
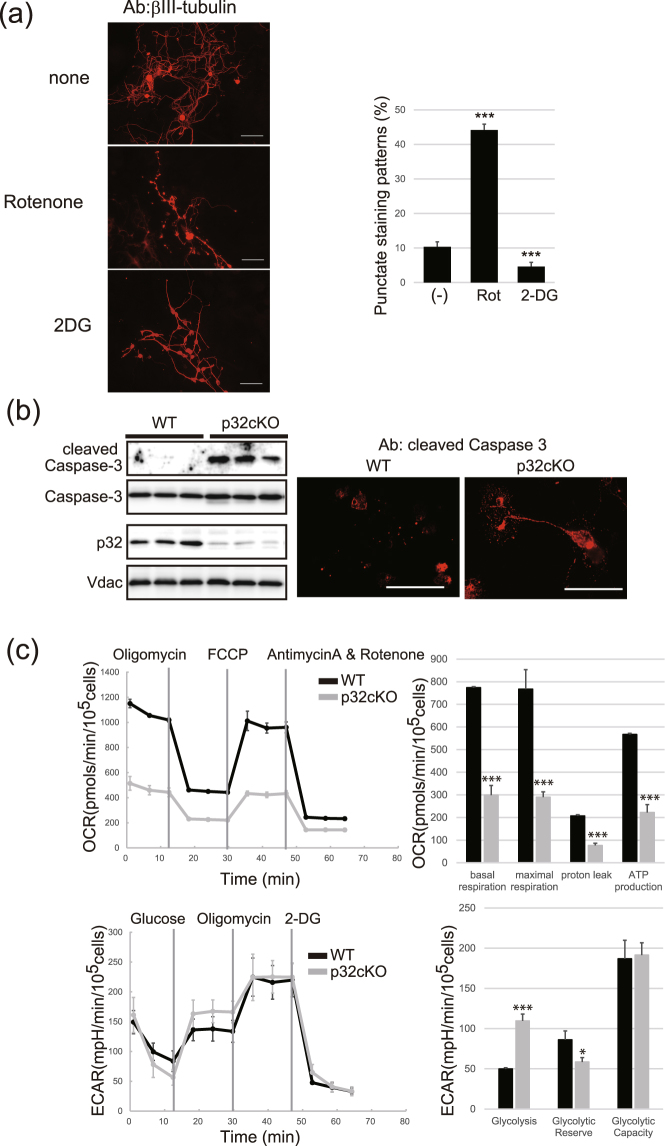
Mitochondrial OXPHOS and axon degeneration. (**a**) Sensitivity of neuron maintenance to rotenone and 2-DG. Primary neurons were incubated for 7 days, and treated with rotenone (1 nM) or 2-DG (10 mM) for another 2 days. Neurons were stained with βIII-tubulin and the number of dot like structures in wild-type neurons was measured. Scale bar = 50 μm. (**b**) Primary neurons were cultured for 7 days and the expression of cleaved Caspase 3 was measured by western blotting (left panel) and immunostaining (right panel); n = 3 independent mouse brain cultures. Increased cleaved Caspase 3 expression and dot-like structures in whole neurites were observed in p32cKO neuron cultures. Scale bar = 50 μm. (**c**) Basal oxygen consumption rate and extracellular acidification rate of wild-type and p32cKO cultures was assessed using the Seahorse Bioscience Flux Analyzer (mean ± SD, n = 3 per group). *p < 0.05, **p < 0.005. ***P < 0.05 vs. WT.

Next we investigated the effects of mitochondrial inhibition during the neurite maintenance process. After 7 days of culture, we treated neurons with rotenone and 2-DG for another 2 days and immunostained with βIII-tubulin. We found dot-like staining after rotenone treatment in neurites and axons, showing that mitochondrial respiratory chain function was essential for neurite maintenance (Fig. 4a). In contrast, 2-DG did not inhibit neurite maintenance. These results indicate that neurite outgrowth was dependent on glycolysis, but that neurite maintenance and oligodendrocyte differentiation were dependent on respiratory chain activity, suggesting to a role for p32 in oligodendrocyte differentiation and neurite maintenance but not neurite outgrowth.

### Activated cleaved caspase 3 in neural axons

Caspase 3 is important not only in apoptosis but also in physiological processes that do not cause cell death. In primary neuron culture, high magnification of single p32cKO neurons demonstrated diffuse cleaved caspase 3 staining in the cytoplasm and neurites, suggesting the accumulation of activated caspase 3 in these compartments (Fig. 4b). We also observed a punctuated pattern of βIII-tubulin in primary neurons (Fig. 3a), suggesting that the dot-like structure of βIII-tubulin might result from activated caspase 3 cleavage, because βIII-tubulin is a caspase substrate.

We further assessed oxygen consumption rate by the mitochondrial electron transport chain (ETC) as an index of cellular energy metabolism with the aid of a Seahorse XF24 extracellular flux analyzer. After normalization, the oxygen consumption rate in the p32cKO primary neuron was significantly decreased compared with that in the wild-type neuron (Fig. 4c). Under these conditions, glycolytic flux, as assessed by the extracellular acidification rate, was analyzed. We observed increased basal glycolysis and decreased glycolytic reserve in the p32cKO brain, suggesting that high glycolysis dependency was due to decreased respiratory chain activity in p32cKO primary cultured neurons (Fig.4c). Seventy-two hours after plating the neuron cell suspension, the p32cKO culture medium was acidified, suggesting that p32cKO neurons are dependent on glycolysis (Supplementary Fig. 7). These data support the hypothesis that p32 depletion resulted in the inhibition of mitochondrial translation and mitochondrial respiratory chain enzymes, and increased glycolysis activity in the p32cKO mouse brain.

### Reduced mTOR signaling

**Figure 5:**
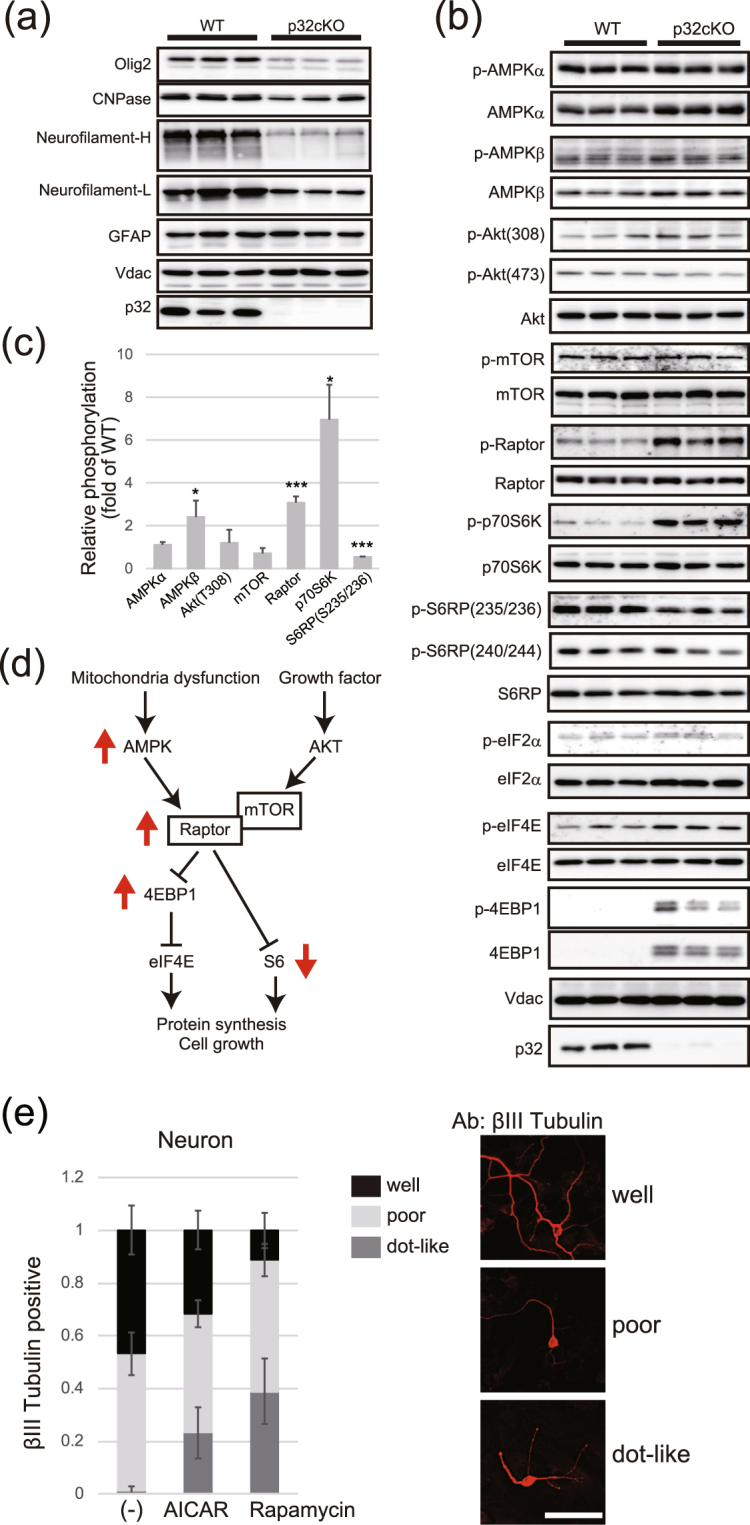
Reduced mTOR activity in the p32cKO brain. (**a**) Immunoblot analyses of 5-week-old brain white matter using antibodies against the indicated proteins; n = 3 mice per genotype. (**b**) Immunoblot analysis of p32cKO brain white matter and quantification of band intensity. (**c**) Integrated density values for each lane were normalized to Voltage-dependent anion channels (Vdac) and are expressed relative to the lowest control value. *P < 0.05, vs. control. (**d**) Schema of the mTOR signaling pathway. Upper arrow indicates upregulation in p32cKO compared with wild-type. (**e**) Sensitivity of neuron maintenance to AICAR and rapamycin. Primary neurons were incubated for 4 days, and treated with AICAR (10 µM) or rapamycin (10 mM) for another 2 days. Neurons were stained with βIII-tubulin and the number of low staining or dot like structures in wild-type neurons was measured. Scale bar = 50 μm.

To evaluate oligodendrocyte differentiation and axonal degeneration, we performed immunoblotting in p32cKO mice with white matter region markers. The levels of Oligo2, CNPase and Neurofilament H protein were significantly reduced in the p32cKO brain, suggesting that oligodendrocytes and axon degeneration occurred in the white matter region (Fig. 5a). AMP kinase (AMPK) activity is involved in mitochondrial biogenesis; therefore, we performed immunolabeling for phosphorylated AMPK and mammalian target of rapamycin (mTOR) pathways in the white matter regions of wild-type and mutant brains. In p32cKO brain extracts, we observed increased phosphorylation of Raptor, AMPKβ, p70S6K and 4EBP1, suggesting that the mTOR pathway was inhibited in the p32cKO brain (Fig. 5b and c). Finally, 4EBP1, which binds to eIF4E and inhibits translation, was also increased in the p32cKO brain, suggesting that cytosolic translation might be inhibited in the p32cKO brain (Fig. 5d). This might contribute to axon degeneration, leading to cerebellar ataxia in the mutant mice. Next we investigated the effects of mTOR signaling during the neurite maintenance process. After 4 days of culture, we treated neurons with AICAR, AMPK activator or rapamycin, mTOR inhibitor for another 2 days and immunostained with βIII-tubulin. We found dot-like staining and axon degeneration after AICAR or rapamycin treatment in neurites, showing that mTOR signaling might be involved in neurite maintenance (Fig. 5e).

### Mitochondrial dysfunction in p32cKO mice activates a maladaptive integrated stress response (ISR)

In an attempt to identify potential disease-causing processes, we carried out gene-expression profiling. qRT-PCR analysis showed the upregulation of a number of genes activated by the integrated stress response (ISR) (e.g., *Gadd34*, *Ddit3*/*Chop*, *Mthfd2*, *Slc7a5 Angptl6* and *Trib3*) in the p32cKO brain (Table 1). Western blot analysis also showed slightly increased phosphorylation of eIF2α in p32cKO white matter compared with controls (Fig. 5b). The maladaptive activation of the ISR downstream of eIF2α phosphorylation in p32cKO white matter region is thus a primary effect of mitochondrial dysfunction in p32cKO.

**Table 1:** Genes with increased or decreased expression in p32cKO.

Symbol	Genebank accession no.	Description	fold change	T test
Gdf15	NM_011819.2	growth differentiation factor 15	85.92	0.00013
Fgf21	NM_020013.4	fibroblast growth factor 21	53.16	0.03050
Cdsn	NM_001008424.2	Corneodesmosin	43.75	0.018
Eif4ebp1	NM_007918.3	eukaryotic translation initiation factor 4E BP 1	16.48	0.00001
Hmox1	NM_010442.2	heme oxygenase 1	13.12	0.02098
Trib3	NM_175093.2	tribbles homolog 3	10.68	0.00026
Atf3	NM_007498.3	activating transcription factor 3	7.33	0.01346
Slc7a5	NM_011404.3	solute carrier family 7	4.95	0.0000004
Otc	NM_008769.4	ornithine transcarbamylase	3.51	0.01438
Acacb	NM_133904.2	acetyl-Coenzyme A carboxylase beta	2.51	0.00055
Sesn2	NM_144907.1	sestrin 2	2.23	0.00011
Igfbp3	NM_008343.2	insulin-like growth factor binding protein 3	2.21	0.02494
Nmnat3	NM_144533.2	nicotinamide nucleotide adenylyltransferase 3	2.06	0.00309
Ddit3	NM_001290183.1	DNA-damage inducible transcript 3	2.02	0.00075
Idh2	NM_173011.2	socitrate dehydrogenase 2 (NADP+), mitochondrial	2.01	0.00397
Mgst1	NM_019946.4	microsomal glutathione S-transferase 1	1.94	0.00025
Gpd1	NM_010271.2	glycerol-3-phosphate dehydrogenase 1 (soluble)	1.89	0.02971
Nqo1	NM_008706.5	NAD(P)H dehydrogenase, quinone 1	1.80	0.03335
Nfe2l2	NM_010902.3	nuclear factor, erythroid derived 2, like 2	1.79	0.00525
Ppp1r15a	NM_008654.2	protein phosphatase 1, regulatory subunit 15 A	1.64	0.05324
Gabarapl1	NM_020590.4	GABA receptor-associated protein-like	0.76	0.04963
Fbxo32	NM_026346.3	F-box protein 32	0.64	0.01779
Hmgcr	NM_008255.2	3-hydroxy-3-methylglutaryl-Coenzyme A reductase	0.66	0.00280
Fabp5	NM_001272097.1	fatty acid binding protein 5	0.57	0.01736
Acyl	NM_001199296.1	ATP Citrate Lyase	0.50	0.00129
Ldlr	NM_001252658.1	low density lipoprotein receptor	0.38	0.00012

List of representative genes whose expression level was 1.5-fold higher or 0.80-fold lower in p32cKO compared with wild type mouse heart. (n = 6 samples per each group).

In p32cKO mice, increased levels of *Gdf15* and *Fgf21* mRNA, novel diagnostic markers for mitochondrial disease^23,24^, were also observed (Table 1). This analysis demonstrated the upregulation of a number of genes activated by the ISR and of mitochondrial disease diagnostic markers in mitochondrial metabolism-disrupted p32cKO mice.

### Reduced levels of sphingomyelin and fatty acid synthesis in p32cKO mice

**Figure 6:**
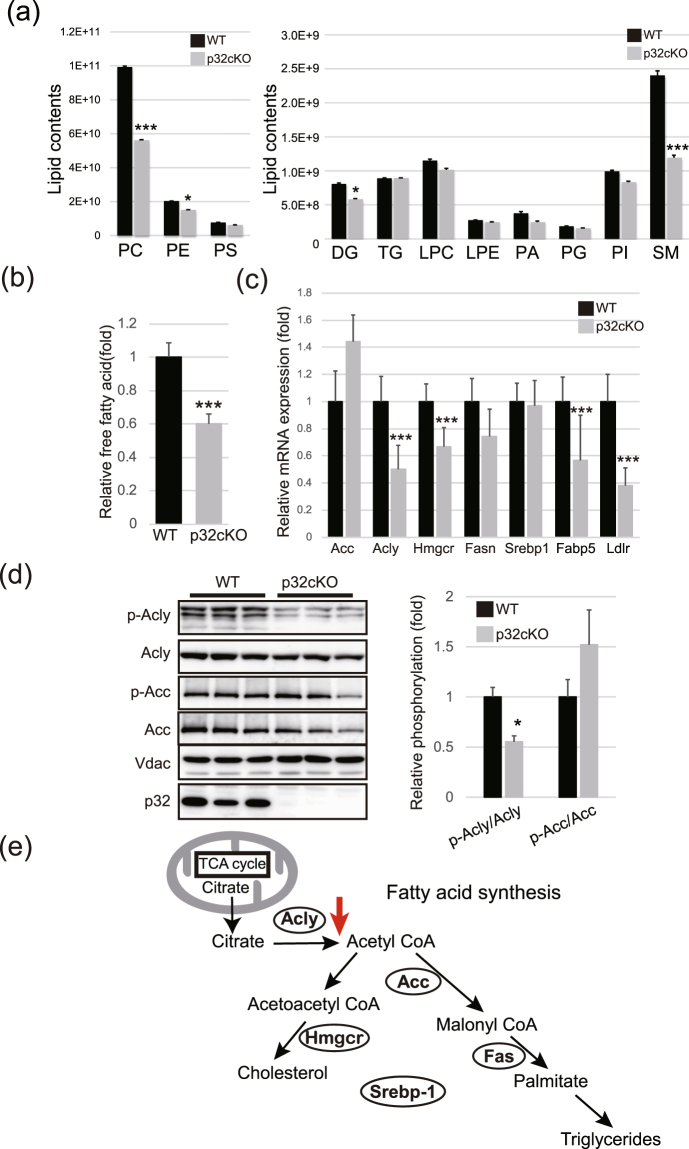
Reduced levels of lipid metabolites and fatty acid synthesis. (**a**) Lipid metabolomics showed reduced levels of phosphatidylcholine and sphingomyelin (n = 3 mice per genotype). PC: phosphatidylcholine, PE: phosphatidylethanolamine, PS: phosphatidylserine, DG: diacylglycerol, TG: triacylglycerol, LPC: lysophosphatidylcholine, LPE: lysophosphatidylethanolamine, PA: phosphatidic acid, PG: phosphatidylglycerol, PI: phosphatidylinositol, SM: sphingomyelin. (**b**) Free fatty acid levels were significantly reduced in 6-week-old p32cKO brain extract. (**c**) qRT-PCR analysis confirmed that Acyl ATP lysase (Acly) is down-regulated in 6-week-old p32cKO compared with control brain. Srebp1, Sterol regulatory element binding transcription factor 1; Acly, ATP citrate lyase; Acc, Acetyl-CoA carboxylase beta; Fas, Fatty acid synthase, Hmgcr: Methylglutaryl-CoA reductase (**d**) Immunoblot analysis and quantification of band intensity in the p32cKO brain; n = 3 mice per genotype. Error bars represent SD; *p < 0.05 (**e**) Schema for the fatty acid synthesis pathway.

Next, we performed lipid metabolomic analysis of the p32cKO mouse brain and analyzed 10 lipid classes by LC-MS. We found that phosphatidylcholine (PC) and sphingomyelin (SM) were significantly reduced in p32cKO mice, leading to hypomyelination and dysfunction of nerve cell membranes (Fig. 6a).

Fatty acid composition was used as an index of the maturation stage of developing brain membranes. We measured the free fatty acid composition in the white matter of wild-type and p32cKO mice. At 5 weeks old, the free fatty acid content was significantly decreased in p32cKO brains (Fig. 6b), suggesting that fatty acid biosynthesis might be impaired in p32-deficient brains. Furthermore, qRT-PCR confirmed that the ATP citrate lysate (*Acly*) gene, which is critically involved in lipid synthesis, was down-regulated in p32-deficient brains (Fig. 6c). Moreover, we found that phosphorylated Acly, which is the active form, was significantly decreased in the p32cKO brain (Fig. 6d). Expression and phosphorylation of acetyl-CoA carboxylase (Acc), which is involved in malonyl-CoA synthesis, were not different between these mice. Acly plays a central role in energy metabolism as it synthesizes acetyl-CoA, a precursor for both fatty acid and cholesterol synthesis (Fig. 6e). These results suggest that *Acly* expression and function might be involved in oligodendrocyte development.

### Metabolomic analysis

**Figure 7:**
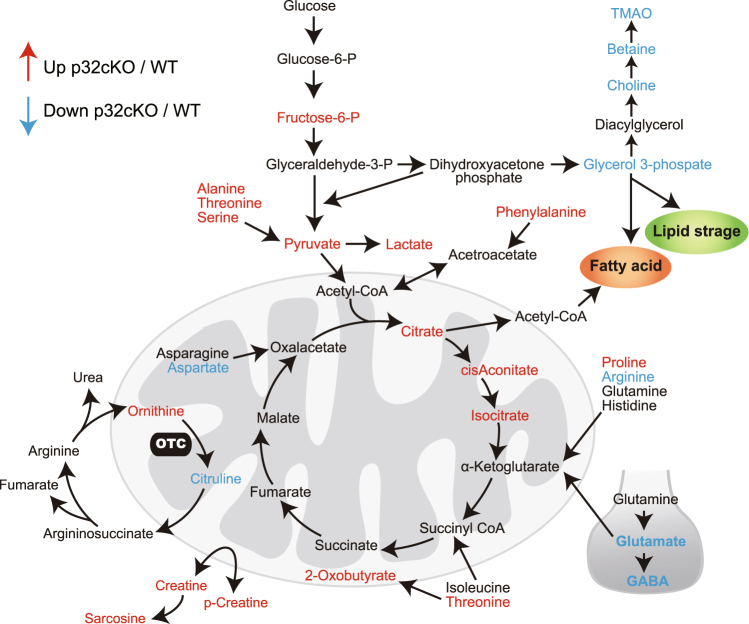
Metabolomic analysis. Metabolomic analysis of the p32cKO mouse brain. Red arrows indicate significantly up-regulated metabolites, and blue arrows indicate significantly down-regulated metabolites in the p32cKO brain compared to wild type.

To examine in more detail the mechanism of p32 deletion-induced metabolic changes in the pathology observed in the p32cKO brain, we performed a comprehensive metabolomic analysis of 6-week-old wild-type and p32cKO white matter extract. In this study, we acquired LC-MS spectra of brain extracts from four paired p32cKO and normal mice. Among the 40 identified differential metabolites contributing most to the discrimination of p32cKO from normal controls, 25 metabolites were increased and 15 were decreased in p32cKO white matter (Fig. 7 and Supplementary Table 1).

The increased levels of lactate, pyruvate, fructose-6-phosphate, creatine and phospho-creatine indicate an increase in glycolysis because of respiratory chain dysfunction in p32-deficient mice. 3-Hydroxybutyrate, a by-product of fatty acid metabolism, was elevated, suggesting reduced ketone usage in p32-deficient neurons (Supplementary Table 1). γ-Aminobutyric acid (GABA), glutamine, glutamate and choline concentrations were decreased in the KO brain, suggesting a deficiency in neurotransmitter cycling, leading to reduced neural synaptic activity. Choline, betaine and TMAO levels were also significantly decreased in the p32cKO mouse brain. Such decreases have been linked to neurological impairment. We found significantly increased levels of five amino acids (alanine, phenylalanine, proline, serine and threonine) and significantly decreased levels of three amino acids (arginine, glutamate and aspartate). These metabolome analyses demonstrated increased glycolysis, ketone usage, altered glutamate metabolism, neurotransmitter signaling and serine and threonine metabolism due to p32 deficiency in mitochondria.

### Decreased ornithine transcarbamylase activity

**Figure 8:**
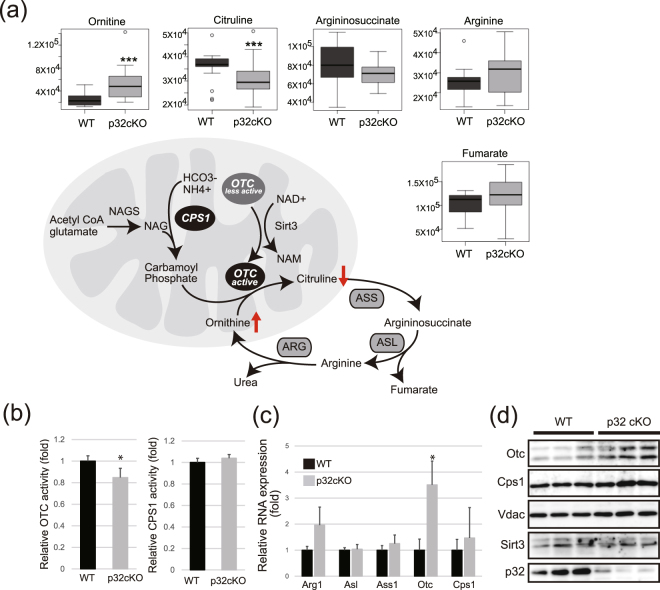
Urea cycle dysfunction. (**a**) Enzymes of the urea cycle. Ammonia from mitochondria is converted to urea in the cytosol, and ornithine is transported back into mitochondria to continue the cycle. Metabolomic analysis showed a significant accumulation of ornithine and a decreased level of citrulline in brain white matter extract of 5-week-old p32cKO versus control mice. (**b**) OTC and CPS activity were measured in brain white matter extracts of 5-week-old p32cKO versus control mice. Error bars represent SD; *p < 0.05. (**c**) qRT-PCR analysis confirms that *Otc* mRNA was upregulated in p32cKO brains. (**d**) Immunoblot analysis and quantification of band intensity show increased Otc levels in p32cKO nerves; n = 3 mice per genotype. Arg1: arginase 1, Asl: Argininosuccinic lyase, Ass1: Argininosuccinate synthetase, Otc: Ornithine carbamoyltransferase, Cps1: Carbamoyl phosphate synthetase 1.

Metabolomic analysis of p32-deficient brains revealed increased levels of ornithine and decreased levels of citrulline metabolites, suggesting that the urea cycle was affected in the p32cKO brain (Fig. 8a). The urea cycle consists of five reactions, two mitochondrial and three cytosolic. We measured the activity and expression of carbamoyl phosphate synthetase 1 (Cps1) and ornithine transcarbamylase (Otc), which are localized in the mitochondrial matrix. In the p32cKO brain, OTC activity, but not CPS1 activity was significantly reduced (Fig. 8b). However, *Otc* mRNA and protein expression were slightly increased in p32cKO mice (Fig. 8c and d), suggesting that Otc might be in an inactive form, possibly because of acetylation. These results suggest that p32 might be involved in urea cycle regulation in brain mitochondria.

## Discussion

In this study, we describe the characterization of p32 nestin-specific knockout mice with disrupted mitochondrial function in neurons, oligodendrocytes and astrocytes. The main finding is that p32 knockout leads to neurological dysfunction and to development of symmetrical and bilateral encephalopathy characterized by spongiosis, demyelination and astrogliosis (Fig. 1). The lesions are restricted to white matter regions that contain oligodendrocytes and neural axons. In primary cultures, p32cKO oligodendrocytes showed reduced differentiation and differentiation of wild-type oligodendrocytes was inhibited by rotenone, suggesting that oligodendrocyte differentiation might depend on respiratory chain activity (Fig. 3). Wild-type and p32cKO primary neurons showed similar neurite outgrowth, which depends on glycolytic activity; however, axonal generation was reduced in p32cKO primary neurons because of respiratory chain dependency (Fig. 4). These result suggested that forebrain sections from p32cKO mice showed spongiform degeneration within the pons, medulla and midbrain, but not in the cerebral or cerebellar cortices (Fig. 1). Moreover, p32-deficient and wild-type astrocytes showed similar degrees of growth (Fig. 3), because maintenance in these cells is less dependent on respiratory chain dysfunction or glycolysis activity. We have identified the main disease processes in oligodendrocytes and axons that contribute to the pathology of mitochondria-related leukoencephalopathy in p32-deficient mice.

To identify the molecular consequences of p32 deficiency in the brain, we showed that respiratory-chain deficiency in the white matter region activates a maladaptive ISR (Table 1). We also demonstrated that p32 depletion reduced OTC activity and changed the expression of fatty acid synthesis genes (Fig. 8). This shift results in depletion of important myelin components, which initially disrupts axon-glial interactions and is a probable driver of subsequent hypo-myelination or demyelination in respiratory chain-deficient-related leukoencephalopathy. Finally, we propose a mechanism by which the mTOR pathway is impaired in p32cKO mice (Fig. 5). Taken together, activation of a maladaptive ISR, mTOR pathway and disruption of lipid metabolism homeostasis secondary to mitochondrial dysfunction might be critical pathological mechanisms in p32-related leukoencephalopathy.

Three weeks after birth, we observed decrease in food intake and movement of p32cKO mice. Before dying, these mice had less visceral fat and higher epilepsy, suggesting that a reduction in food intake and severity of visceral fat and disease progression resulted in premature death of p32cKO mice. Next-generation sequencing analysis of the exome identified p32 as a new disease-causing gene in mitochondrial respiratory chain disorders^12^. Consistent with these observations from *in vitro* studies, p32 knockout mice resulted in an embryonic lethal phenotype caused by reductions in respiratory chain activity and OXPHOS^9^. In this study, we generated the first mouse model (p32cKO) with disrupted mitochondrial function specifically in neurons and glial cells that can be used to interrogate the contribution of brain mitochondrial impairment to neuropathy. In this mouse model, we identified several metabolites related to mitochondrial dysfunction, suggesting that these metabolites might be useful as diagnostic markers of mitochondrial related neurodegenerative disease.

Spongiform degeneration is caused by extensive vacuolization of neuronal cells, which is typically associated with brain damage induced by prions^25^. The underlying mechanism of such vacuolization is not well understood, but mitochondrial dysfunction has been suggested to be responsible for spongiform degeneration in several disease models^26,27^. Canavan disease (CD) is a globally occurring spongiform leukodystrophy associated with mutation of the aspartoacylase gene, which is highly expressed in oligodendrocytes^28^. In this study, we found signs of spongiform degeneration in the white matter of p32cKO mice as early as 2 weeks of age (Supplementary Fig. 2), preceding the loss of oligodendrocyte cells. Because oligodendrocyte maturation occurs at P10, it is possible that the first target of p32-deficient leukoencephalopathy is oligodendrocyte differentiation.

Aerobic metabolism is the dominant metabolic pathway during early postnatal development when lipids and proteins are needed for the processes of axonal elongation, synaptogenesis and myelination^19^. Furthermore, aerobic metabolism is likely to continue in adults to supply components for activity-related changes at the synapse and for the turnover of structural components of neurons. Conversely, oxidative phosphorylation appears to be the main metabolic support for synaptic transmission and, therefore, this pathway seems to be more dominant in brain structures. In our mouse model, we identified poor synapse structure and decreased levels of metabolite, such as glutamate and GABA (Fig. 7), suggesting that mitochondrial function is involved in synaptic activity. Furthermore, it was recently found that aerobic glycolysis is necessary and sufficient for fast axonal transport along the length of the axon to nerve terminals, suggesting that aerobic glycolysis is also important as an energy source for the delivery of molecules necessary for axonal elongation^29^.

Glial cells of the blood–brain barrier specifically take up sugars, the metabolism of which relies on glycolysis, which is essential for neuronal survival^20^. We also found that mitochondrial respiratory chain function in oligodendrocytes is essential for myelination and differentiation, and that glycolysis in oligodendrocytes is also involved in oligodendrocyte differentiation (Fig. 3), suggesting that mitochondrial function is involved in oligodendrocyte differentiation. However, we did not identify the metabolic interaction between oligodendrocytes and neurons in this nestin-cre system. In the near future, we will investigate the metabolic interaction between them using individual p32 knockout strains.

We observed increased Raptor phosphorylation in p32cKO mice, which reduced mTOR activity, leading to inhibition of axon maintenance (Fig. 5). We also observed increased 4EBP phosphorylation and increased ER stress response gene expression in p32cKO mice. It was reported that mTOR regulates oligodendrocyte differentiation at the late progenitor to immature oligodendrocyte transition^30^. Mutations of a translation initiation factor, eIF2B lead to leukoencephalopathy with vanishing white matter, an inherited chronic-progressive fatal brain disease^31^. These patients show morphological abnormalities and lack of function, leading to insufficient myelin deposition, loss of myelin and insufficient gliosis for the degree of white-matter damage. Activation of the unfolded-protein response has also been confirmed in brains of patients with vanishing white matter, with increased activity of phosphorylated eIF2α, activating transcription factor-4, and CHOP, suggesting that inappropriate activation of the unfolded-protein response and a decrease in the translation pathway is important in the pathophysiology of leukoencephalopathy^32^.

In primary neuron culture, p32cKO neurons demonstrated diffuse cleaved caspase 3 staining in the cytoplasm and neurites (Fig. 4) and a punctuated pattern of βIII-tubulin in primary neurons (Fig. 3), suggesting that the dot-like structure of βIII-tubulin might result from activated caspase 3 cleavage. We did not observe apoptosis in p32cKO brain region by using TUNEL assay (data not shown) and cell body of neuron is intact, suggesting that activate caspase-3 might be involved in degradation of substrate such as βIII-tubulin in neuron but not in caspase dependent cell death in p32cKO brain.

It was reported that 4EBP1 protein and its mRNA are upregulated and transported to dendritic domains in RNA granules upon neuronal activation^33^. Local protein synthesis in axons is a critical component of synaptic plasticity. In synaptic plasticity, mTOR serves as a primary trigger for the initiation of cap-dependent translation via phosphorylation of 4EBPs and S6Ks. Here, we observed reduced mTOR activity and 4EBP1 upregulation, leading to an adaptive response to mitochondrial dysfunction. Activation of an ISR promotes a cellular stress-resistant state by global attenuation of protein synthesis, which reduces the ER load and diverts amino acids from energetically costly protein synthesis to other metabolic pathways^34^.

We found several metabolites that are altered in the p32cKO mouse brain. Neurotransmitters, such as GABA and glutamine, were decreased in p32cKO brains, suggesting a deficiency in neurotransmitter cycling, leading to reduced neural synaptic activity. Choline, betaine and TMAO levels were also decreased in the p32cKO mouse brain. Previous studies reported decreased choline levels in animal models and epilepsy patients^35,36^. 3-Hydroxybutyrate is a metabolite produced from the degradation of glyceride and fatty acids. Ketone bodies can, and do in some naturally occurring conditions, substitute, at least in part, for glucose^37^. This elevated 3-hydroxybutyrate level is presumed to be produced as a result of high levels of glyceride mobilization in response to higher energy demands in the brain. Disturbed aspartate and alanine metabolism has been reported in the dorsal prefrontal cortex of schizophrenia patients^38^. Glycerol-3 phosphate (Gly3P) is an important component of glycerol metabolism and should still be available for fatty acid esterification. These metabolites might be useful as diagnostic markers of mitochondrial disease.

Deletion of p32 in neural progenitor cells led to several typical neuropathies, including progressive axonopathy and spongiform degeneration, as well as increased demyelination of oligodendrocytes. These cellular defects resulted in axon degeneration, development of ataxia, and early death of p32cKO mice. Further studies are required to resolve the potential contributions of these and possibly other signaling pathways in mediating myelination defects in p32cKO mice.

In summary, our study suggests p32 is important for mitochondrial function and that mitochondrial function is essential for oligodendrocyte differentiation and axon support. These results provide novel insight into the pathogenesis of leukoencephalopathy diseases and the role of mitochondria in prevention of these diseases. The p32 conditional KO mouse may be a useful mouse model for future studies on the mechanisms of leukoencephalopathy and ataxia as well as for identifying promising diagnostic markers for these diseases.

## Materials and Methods

### Mating of transgenic mice

Mouse experiments and protocols were performed in accordance with the guidelines of the animal ethics committee of Kyushu University Graduate School of Medicine, Japan (#A29-052-0). All experimental procedures confirmed to the Guide for the Care and Use of Laboratory Animals, Eighth Edition, updated by the US National Research Council Committee in 2011 and approved by the guidelines by the Kyushu University Animal Care and Use Committee. Mice were housed in a 12-h light–dark cycle at 22 °C and had free access to rodent diet and water. p32^loxP/loxP^ mice in a pure C57BL/6 background^9^ were crossed to nestin-Cre mice (B6.Cg-Tg(Nestin-cre)1Kln/J) (The Jackson Laboratory, Bar Harbor, ME) also in a pure C57BL/6 background. Compound heterozygotes (nestin-Cre^+/−^, p32^+/loxP^) were then crossed to homozygous p32loxP/loxP mice to generate p32cKO mice (nestin-Cre^+/−^, p32^loxP/loxP^) and their control littermates (nestin-Cre^−/−^, p32^loxP/loxP^). Genotyping for p32 and Cre alleles was performed by PCR analysis of tail DNA, essentially as described previously^9^.

### Immunofluorescence, immunohistochemistry and electron microscopy

Mice at different stages of disease were anesthetized with an overdose of sevoflurane. After exsanguination under deep anesthesia, tissues were fixed in 4% paraformaldehyde and paraffin-embedded coronal sections prepared for histological staining with Kluver–Barrera (KB) or H&E and for enzyme immunohistochemistry. Immunohistochemistry and immunofluorescence were performed as previously described^9,11^. For electron microscopy, 50- to 100-nm-thick ultrathin sections were prepared, stained with uranyl acetate and lead citrate and photographed with a JEOL (Akishima, Japan) 1200 electron microscope.

### Western blotting

Briefly, brain tissues were frozen in liquid nitrogen immediately upon dissection. Tissues and primary cells were lysed with lysis buffer, homogenized by sonication, and then subjected to immunoblotting as described previously^39^.

### Preparation and analysis of neuron, oligodendrocyte and astrocyte cultures *in vitro*

All experiments were performed in accordance with the guidelines of the genetically engineering committee of Kyushu University Graduate School of Medicine, Japan (#27-16-63). Neuronal cell cultures were prepared from brains of P2 mice. Isolated brains were cleared of meninges and cut into 0.5-mm^3^ pieces. Tissues were incubated for 15 min at 37 °C in 0.02% trypsin and cells were then dissociated to single cells using a Nerve-Cell Dissociation media Kit (Sumitomo Bakelite, Tokyo, Japan). The resulting neuronal cells were suspended in pre-warmed primary neuron basal medium (PNBM, Lonza, Walkersville, MD) or primary neuronal growth medium (PNGM, SingleQuots Lonza) supplemented with NGF (100 ng/ml).

Purified primary cultures of oligodendroglial lineage from 0- to 2-day-old mice were prepared by a sorting procedure. Briefly, P2 wild-type and p32cKO brains were dissociated to produce a single cell suspension. Oligodendroglial cultures were then purified to a more than 90% A2B5-positive O4-negative and glial fibrillary acidic protein-negative cell population which corresponds to the Oligodendrocyte Progenitor Cell (OPC) developmental stage. The OPC cultures were expanded for up to four passages in culture medium containing B104 neuroblastoma conditioned medium, bovine FGF2, and human recombinant PDGFAA.

For the analysis of morphological differentiation, cells were classified to two morphological categories: simple, bipolar or stellate cells having short primary branches; or complex morphology, cells having very long primary branches with tertiary branches^40^. In this experience, differentiated cells were classified to complex morphology which showed the long tertiary branches with CNPase staining.

### Mitochondrial isolation, complex activity

Mitochondrial isolation and respiratory complex activity assays were performed as described previously^9^.

### Seahorse XF24 flux analyzer

The Seahorse XF24 Flux analyzer (Seahorse Biosciences, Billerica, MA) was used to determine the metabolic profiles of neurons or oligodendrocytes. Seahorse XF24 microplates were seeded with 4 × 10^4^ cells/well and incubated at 37 °C for approximately 4 days. Basal oxygen consumption rate and extracellular acidification rate were measured in the Seahorse XF24 Flux analyzer. Additional measurements were performed after injection of four compounds affecting bioenergetics: oligomycin 0.75 μM, carbonyl cyanide 4-trifluoromethoxyphenylhydrazone (FCCP) 500 nM, 2-deoxyglucose (2-DG) 100 mM, and rotenone 1 μM (all from Sigma-Aldrich). Upon completion of the Seahorse XF24 Flux analysis, cells were trypsinized, counted, and the results were normalized to the number of cells. Statistical analysis was performed using the t-test.

### RNA preparation and qRT-PCR

Total RNA was isolated after homogenization (of the white matter region) using an mRNeasy Minikit (Qiagen) according to the manufacturer’s protocol and as described previously^9^.

### Free fatty acid quantification

Brain FFA content was measured using the FFA quantification kit from BioVision following the manufacturer’s instructions. Fluorescence was measured using Corning black 96-well polypropylene assay plates and the Microplate Multimode Reader with green fluorescence module (Ex 525 nm; Em 580–640 nm). FFA concentrations were calculated using a standard curve for palmitic acid ranging from 0 to 0.02 nmol/µL.

### Urea cycle enzyme activity

Measurement of carbamyl phosphate synthetase 1 (CPS1) and ornithine transcarbamylase (OTC) activities in mouse brain was carried out as described previously^41,42^. The reaction was initiated by addition of liver lysates to the reaction mixture. The reaction mixture contained 50 mM Tris-HCl pH 8.0, 2.5 mM phosphoenopyruvate, 0.2 mM NADH, 30 mM NH_4_Cl, 100 mM KHCO_3_, 5 mM ATP, 10 mM MgSO_4_, 10 mM N-acetylglutamate, 15 U/ml pyruvate kinase/lactate dehydrogenase (Sigma-Aldrich). The reactions were performed at 37 °C and the decrease in absorbance at 340 nm was monitored. The initial velocity of the reaction was calculated to determine the CPS1 activity.

To measure OTC activity, 2–10 μg of total cellular protein were added to 700 μL of reaction mixture (5 mM ornithine, 15 mM carbamyl phosphate, and 270 mM triethanolamine, pH 7.7) and incubated at 37 °C for 30 min. Reactions were stopped by adding 250 μL of 3:1 phosphoric acid/sulfuric acid (by volume). Citrulline production was then determined by adding 50 μL 3% 2,3-butanedione monoxime, incubating at 95–100 °C in the dark for 15 min, and measuring absorbance at 490 nm.

### Statistical analysis

All values are expressed as the mean ± SD, and if no units are specified, values are expressed as percent of control. If not stated otherwise, p values were determined by unpaired, two-tailed Student’s t tests. All statistical analyses were performed using JMP13. p values are designated as ∗p < 0.05, ∗∗p < 0.01, and ∗∗∗p < 0.005; n.s., non-significant (p > 0.05).

### Extraction of glycerophospholipids from mouse brain and LC-MS analysis

We employed a modified Bligh and Dyer procedure^43^. Briefly, after removal and freezing in liquid nitrogen, a lump of mouse brain (100–150 mg) was crushed in a MultiBeads Shocker (Yasui Kikai, Japan) at 2,000 rpm for 10 seconds. Phospholipids were then extracted with 1 ml of ice-cold 0.1 N HCl-Methanol (1:1, v/v). After vortexing for 20 seconds, 500 μl ice-cold chloroform was added. Samples were again vortexed for 20 seconds and centrifuged at 10,000 × g for 1 min at 4 °C. The lower organic phase was isolated and dried using a miVac DUO concentrator (GeneVac). The resulting lipid film was dissolved in 150 μl methanol-chloroform (9:1, v/v) and 1 μl ammonium hydroxide solution was added prior to LC-MS analysis.

### LC-MS analysis

Brain extracts were separated using high-performance LC (HPLC) on a Kinetex C8 column (150 × 2.1 mm, 1.7-μm particle size, Phenomenex, CA) coupled with a triple quadrupole mass spectrometer LCMS-8040 (Shimadzu, Japan). The mobile phase consisted of solvent A (10 mM ammonium formate) and solvent B (acetonitrile), and the column oven temperature was 53 °C. The gradient elution program was as follows: a flow rate of 0.2 mL/min: 0–1 min, 2% B; 1–2 min, 2–67.5% B; 2–20 min, 67.5–92.5% B; 20–32 min, 92.5% B; 32–33 min, 92.5–100% B; 33–48 min, 100% B, 48–49 min, 100–2% B; and was maintained at 2% B until 55 min. For electrospray ionization (ESI), the ionization parameters were as follows; drying gas flow rate, 10 L/min; nebulizer gas flow rate, 2 L/min; CDL temperature, 150 °C; DL temperature, 250 °C; and heat block temperature, 400 °C. Detection of phosphatitylcholine (PC) and phosphatidylethanolamine (PE) was performed in positive ionization mode by precursor ion scanning of a fragment ion of *m/z* 184 and by neutral loss scanning of 141 Da, respectively. On the other hand, phosphatidylserine (PS), phosphatidylinositol (PI), and phosphatidic acid (PA) were detected in negative ionization mode by neutral loss scanning of 87 Da and precursor ion scanning of fragment ions of m/z 241 and 153, respectively. The other MS parameters of collision energy (CE) and *m/z* scan range were as follows; PC (−20, 200–1000); PE (−25, 200–1000); PS (29, 200–1000); PI (45, 200–1000); PA (50, 200–1000).

## Electronic supplementary material

Supplemental file
