# Supplementary material for: Neural-specific deletion of mitochondrial p32/C1qbp leads to leukoencephalopathy due to undifferentiated oligodendrocyte and axon degeneration
Source: Sci Rep. 2017 Nov 9;7:15131. doi: 10.1038/s41598-017-15414-5 (PMC5680297; doi:10.1038/s41598-017-15414-5)
Supplement: Supplementary file 1 — Supplemental file [file 41598_2017_15414_MOESM1_ESM.pdf]

Neural-specific deletion of mitochondrial p32/C1qbp leads to leukoencephalopathy due to undifferentiated oligodendrocyte and axon degeneration

Mikako Yagi[1], Takeshi Uchiumi[1], Noriaki Sagata[1], Daiki Setoyama[1], Rie Amamoto[1,2], Yuichi Matsushima[1] and Dongchon Kang[1]

[1]Department of Clinical Chemistry and Laboratory Medicine, Graduate School of Medical Sciences, Kyushu University, 3-1-1, Maidashi, Higashi-ku, Fukuoka 812-8582, Japan.
[2]Department of Nutritional Sciences, Faculty of Health and Welfare, Seinan Jo Gakuin University, 1-3-5 Ibori, Kokurakita-ku, Kitakyushu 803-0835, Japan

*Correspondence to: Takeshi Uchiumi: Department of Clinical Chemistry and Laboratory Medicine, Kyushu University Graduate School of Medical Sciences, 3-1-1, Maidashi, Higashi-ku, Fukuoka 812-8582, Japan.
Tel: (+81) 92-642-5748; Fax: (+81) 92-642-5772
E-mail: uchiumi@cclm.med.kyushu-u.ac.jp

Supplemental Figure 1

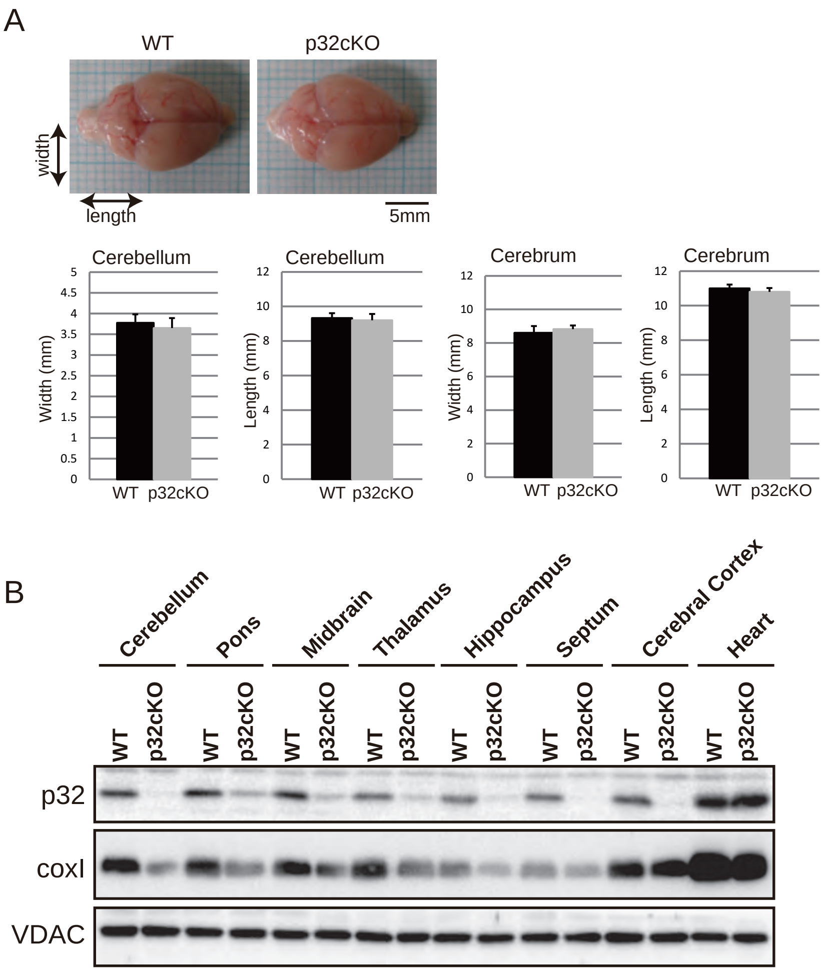

Supplementary Fig. 1

(A) The brain length and width of control and p32nesKO mice of control and p32cKO mice at 6 weeks old. N = 3 independent genotype. Error bars represent SEM. Scale Bar = 5mm.

(B) Immunoblot analyses of various brain region at 5 weeks mouse brain and heart with the use of antibodies against p32, CoxI and VDAC respectivity.

Supplementary Figure 2

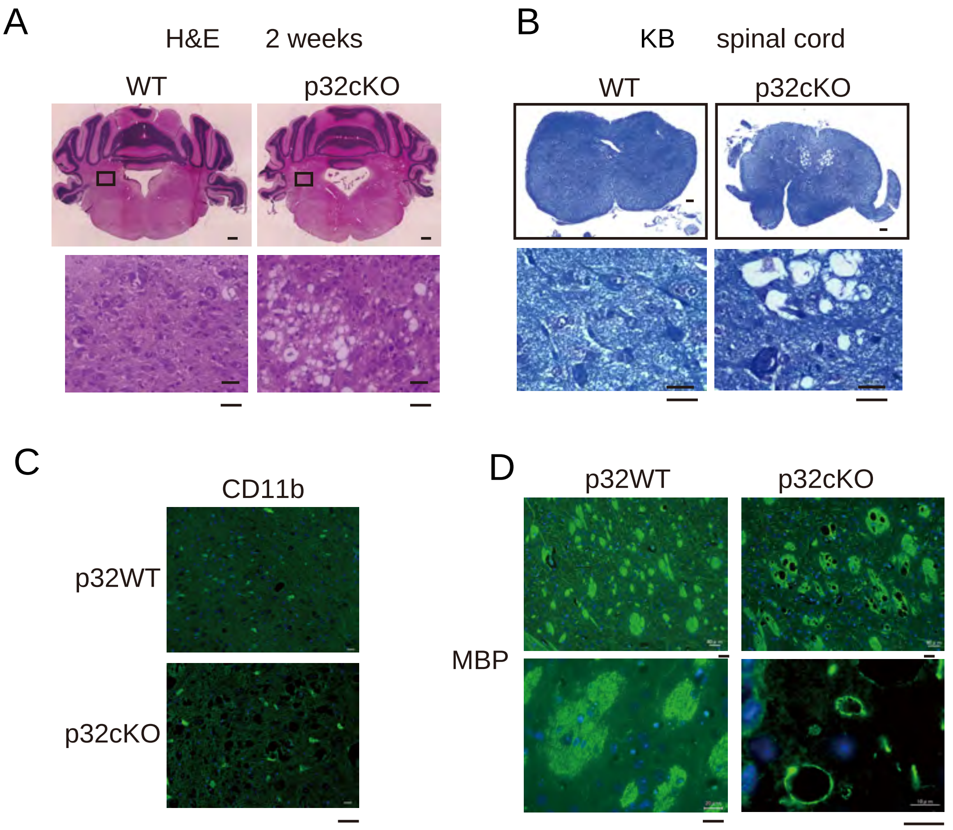

Supplementary Fig. 2

(A) Histological analysis of sagittal sections of cerebellum from control and p32cKO mice at 2 weeks old. The coronal cerebellar sections were stained by hematoxylin & eosin (HE) staining. Lower panel show high magnification view of areas respectively. The vacuoles were observed in the midbrain, middle pons, and medulla in p32cKO. Bars = 200 (upper) and 20 µm (lower).

(B) Histological analysis of sagittal sections of spine from control and p32cKO mice at 5 weeks old. The coronal spinal sections were stained by Kluver-Barrera (KB) staining.

(C)(D) Cerebellum sections from WT and p32cKO mice at 6 weeks of age were analyzed by immunohistochemistry with (C) CD11b for microglia and (D) myelin basic protein (MBP) for oligodendrocyte. Scale bars = 10 µm.

Supplementary Figure 3

A

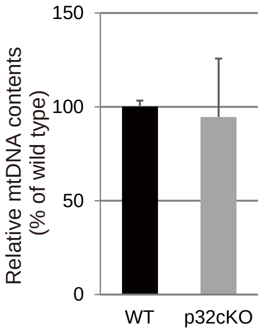

B

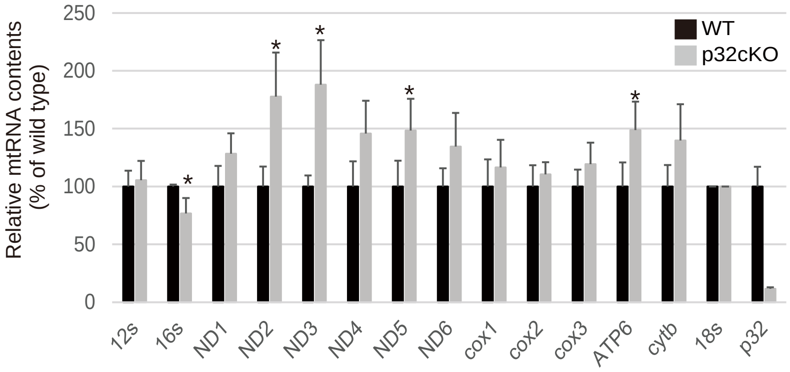

Supplementary Fig. 3

(A) Mitochondrial DNA copy number and RNA contents (B) in brain were analysed by qRT-PCR. Total DNAs and RNA prepared from mouse brain at 5 weeks old were used as the template.

Supplementary Figure 4

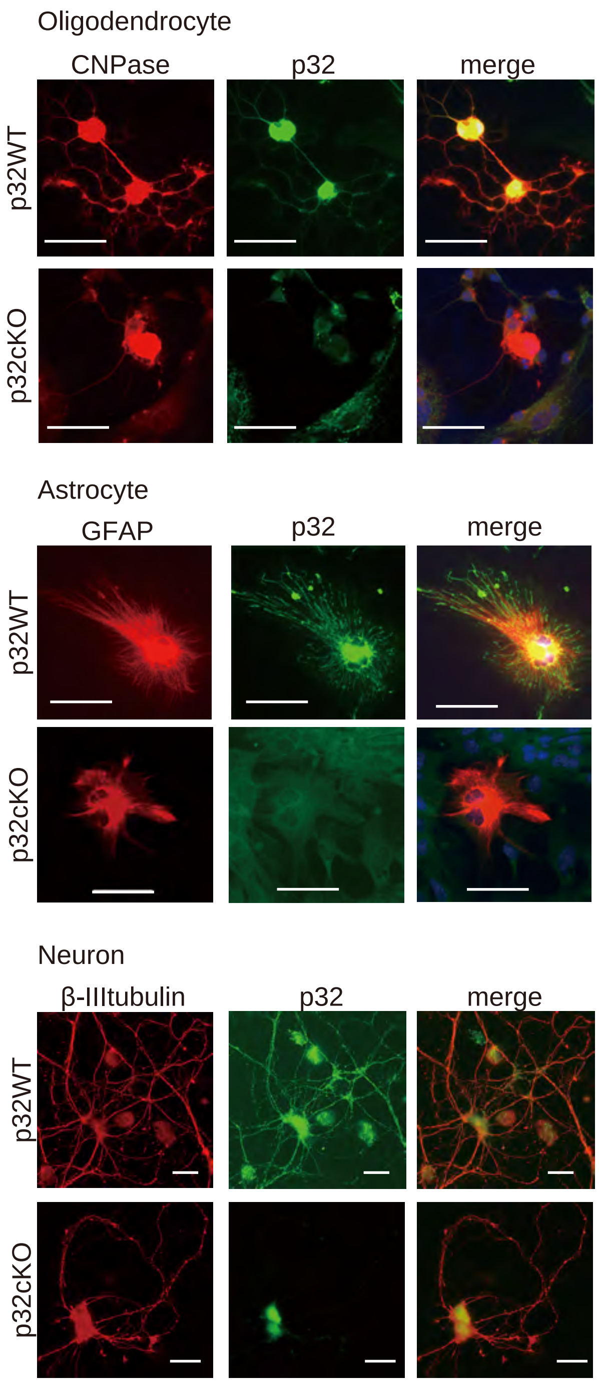

Supplementary Fig. 4

Characterization of oligodendrocyte differentiation, astrocyte growth, neurite outgrowth, and neurite maintenance in cortical cultures during 1~2 weeks. Cultures in wild and p32NesKO were plated on indicated days and reacted with indicated antibody with p32 and/or βIII-Tubulin for neurite, neurofilament for axon generation, CNPase for oligodendrocyte and GFAP for astrocyte. In p32cKO neuron and glia showed that no expression of p32 protein in these primary cells. Scale bars = 50 μm.

Supplemental Figure S5

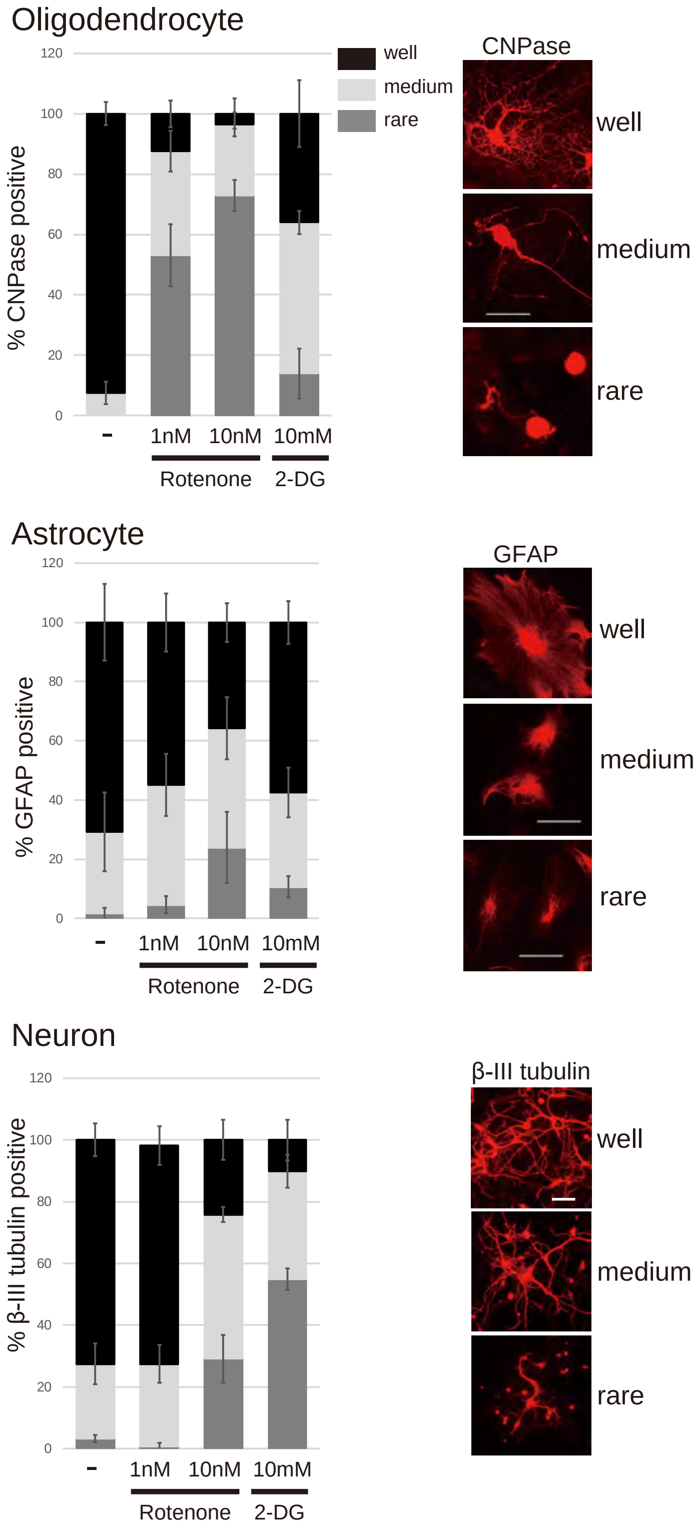

Figure S5

Sensitivity to rotenone and 2DG in oligodendrocyte differentiation and neurite outgrowth. Isolated oligodendrocyte, neuron and astrocyte were treated with complex I inhibitor Rotenone and 2-Deoxy Glucose (2DG). After 7 days culture, cells were staining with each marker and measured the ratio of oligodendrocyte differentiation, neurite outgrowth and astrocyte differentiation. *$p < 0.05$, **$p < 0.005$. Scale bar= 50 μm.

Supplementary Figure 6

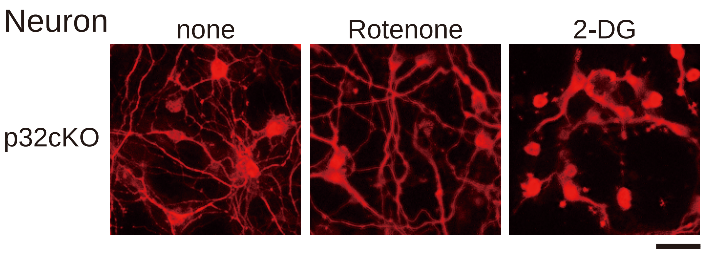

Supplementary Fig. 6

Sensitivity to Rotenone and 2DG in neurite outgrowth. Isolated neuron from p32cKO were treated with complex I inhibitor and 2DG. After 7 days, neuron were staining with βIII Tubulin.

Supplementary Figure 7

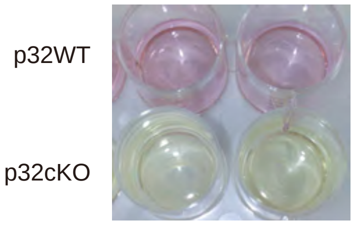

Supplementary Fig. 7

Isolated primary neuron were incubated with DMEM medium for 5 days. p32cKO medium show change color because of increased lactate.

All Western blot -1

Figure 2E

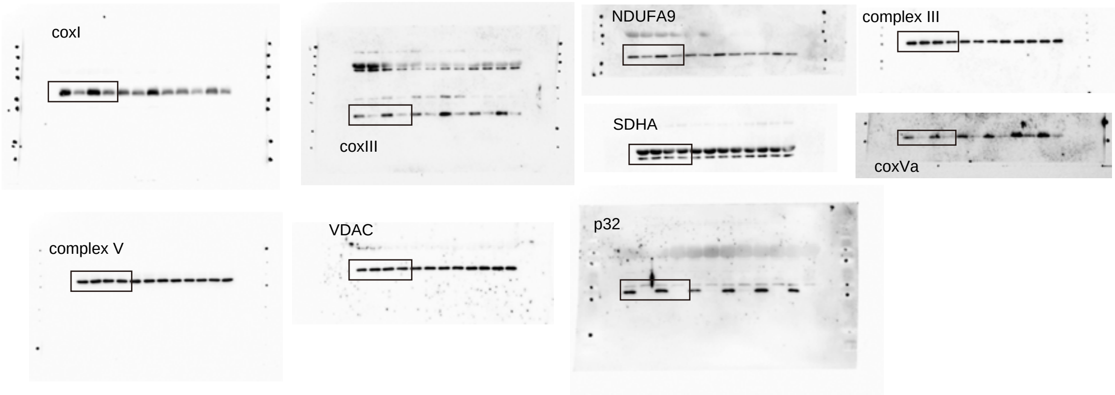

Figure 4B

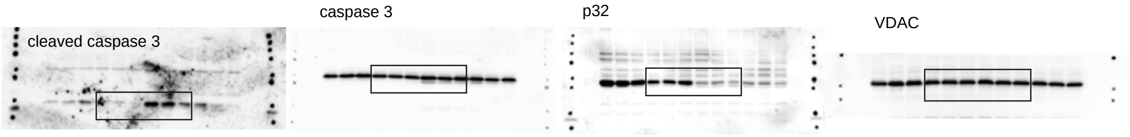

Figure 5A

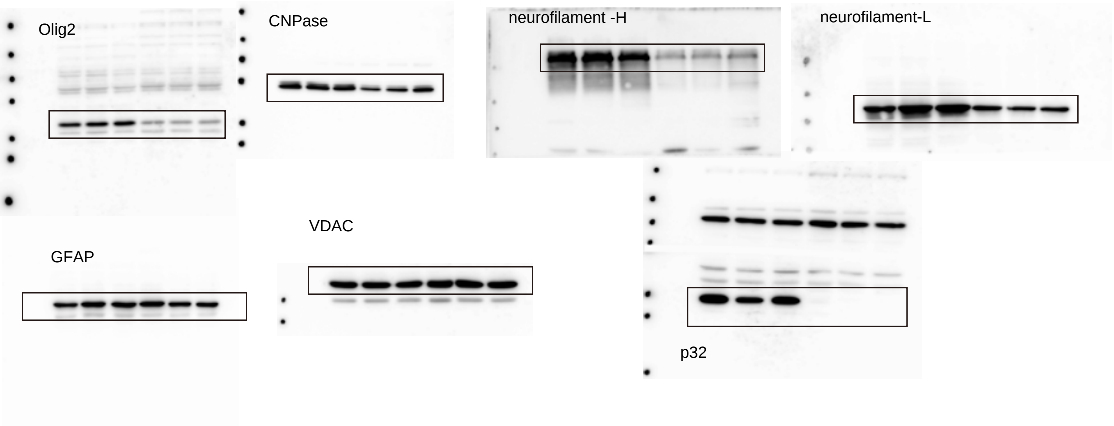

Figure 5B

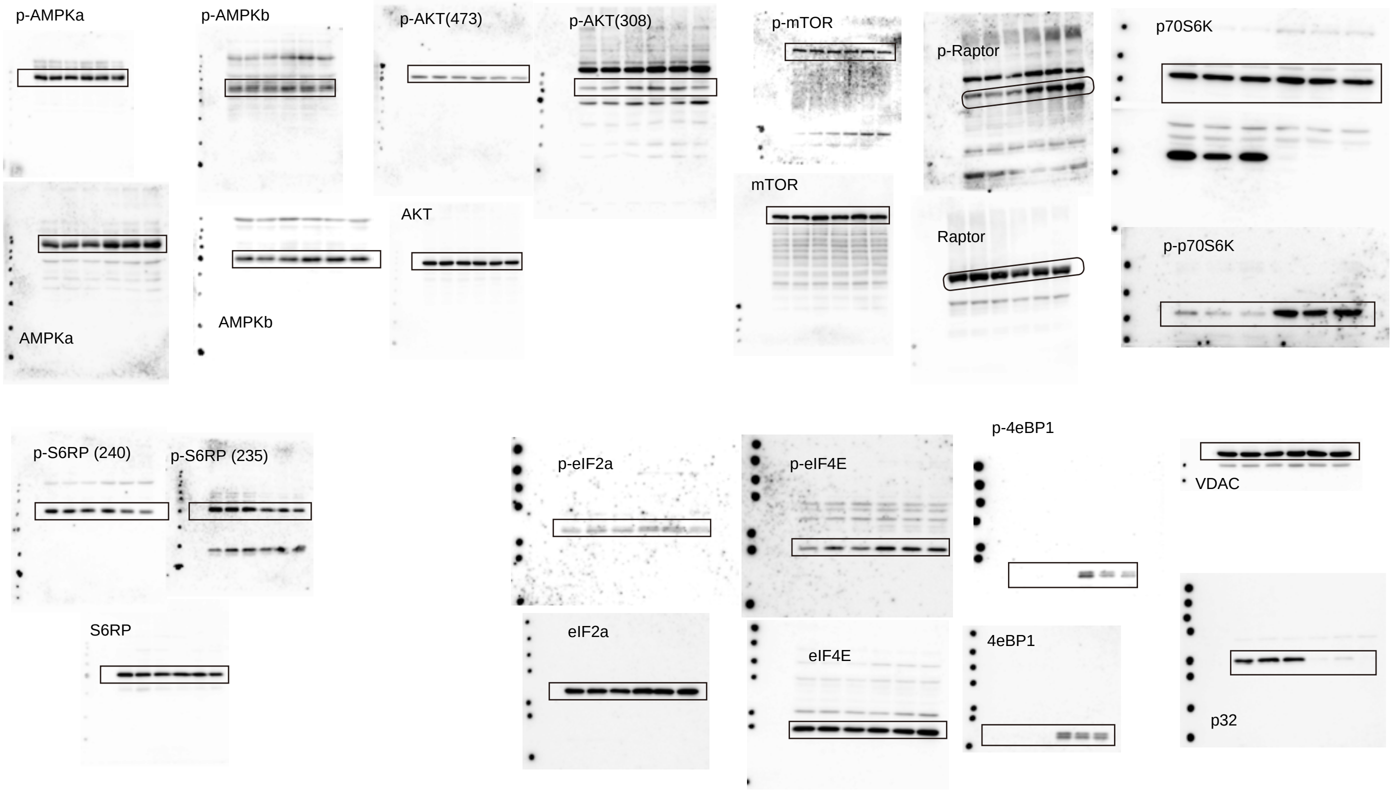

All Western blot -2

Figure 6D

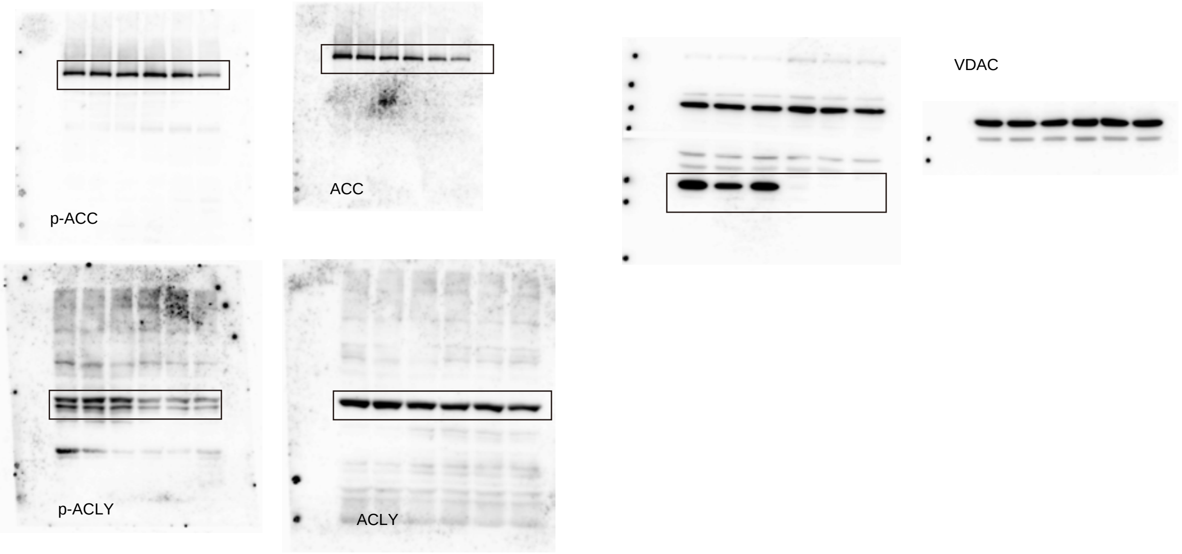

Figure 8D

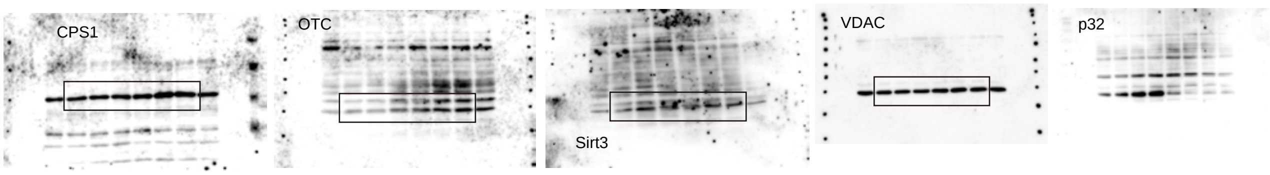

Supplemental Figure 1

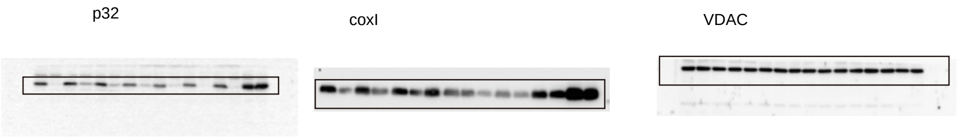

SUPPLEMENTAL Information

Neural-specific deletion of p32/C1qbp leads to Leukoencephalopathy due to undifferentiated oligodendrocyte and axon degeneration

By Mikako Yagi, Takeshi Uchiumi, Noriaki Sagata, Daiki Setoyama, Rie Amamoto, Yuichi Matsushima and Dongchon Kang

SUPPLEMENTAL EXPERIMENTAL PROCEDURE

Mating of transgenic mouse

Mouse experiments were performed in accordance with the guidelines of the animal ethics committee of Kyushu University Graduate School of Medicine, Japan. Mice were maintained with rodent diet and water available ad libitum with 12-h light–dark cycle at 22 °C. p32loxP/loxP mice in a pure C57BL/6 background (Yagi et al., 2012) were crossed to nestin-Cre mice (The Jackson Laboratory (Bar Harbor, ME)) also in a pure C57BL/6 background. Compound heterozygotes (nestin-Cre+/−, p32+/loxP) were then crossed to homozygous p32loxP/loxP to generate the p32NesKO mice (nestin-Cre+/−, p32loxP/loxP) and their control (ctrl) littermates (nestin-Cre−/−, p32loxP/loxP). Mice genotyping for p32 and Cre alleles were performed by PCR analysis of tail DNA, essentially as described previously (Yagi et al., 2012).

Immunofluorescence and immunohisotochemistry

Mice at different stages of disease were anesthesized with an overdose of seboflurane. After exsanguination under deep anesthesia, removed tissue sections were fixed in 4% paraformaldehyde to obtain paraffin-embedded coronal sections for histological staining with luxol fast blue (KB) or H&E, and for enzyme immunohistochemistry.

Immunohistochemistry was performed using a Histofine streptavidin-biotin-peroxidase Kit (Nichirei, Tokyo, Japan). Briefly, the brain tissues were all coronal sectioned at 6 μm and then used for immunofluorescence or immunohistochemistry with primary antibodies. After all staining, sections were mounted in the water-soluble mounting medium (Mowiol) with DAPI (Vector Laboratories) for microscopic visualization (BZ-9000, Keyence, Osaka, Japan).

Fluorescence double immunostaining was performed using combinations of rabbit anti-nestin antibody (Santa Cruz Biotechnology, Inc.) with mouse monoclonal antibody H-4. Cryosections were treated with PBS containing 0.05% Tween 20 (Sigma), 1% bovine serum albumin (BSA) (Sigma), 0.1% sodium azide, goat antimouse IgG (Cappel), and 5% horse serum for blocking. After incubation in a combination of

primary antibodies for 1 h at room temperature, the sections were thoroughly washed with PBS. They were then incubated with FITC–anti-rabbit IgG antibody (Abcam, Tokyo, Japan) or biotin–anti-mouse IgG antibody (Rockland) and avidin-Alexa Fluor 568 (Molecular Probe) for 30 min at room temperature.

Transmission Electron Microscopy

For electron microscopy, fifty- to one hundred-nm-thick ultrathin sections were prepared, stained with uranyl acetate and lead citrate, and photographed with a JEOL (Akishima) 1200 electron microscope. All nerves underwent qualitative assessment of neural architecture followed by detailed histomorphometric analysis carried out as previously described (Hunter et al., 2007). The samples were fixed by perfusion fixation with 2 % paraformaldehyde (GA) in 0.1 M cacodylate buffer pH 7.4. And then, the samples were fixed with 2 % PFA, 2 % GA, 0.5 % tannic acid in 0.1 M cacodylate buffer pH 7.4 at 40°C for 2h. After this fixation, the samples were rinsed 4 times with 0.1 M cacadylate buffer for 15 min each, followed by post fixation with 2 % osmium tetroxide (OsO4) in 0.1 M cacodylate buffer at 40°C for 2h. The samples were dehydrated through a series of graded ethanol (50%, 70%, 90%, 100%).The schedule was as follows: 50% and 70% for 30 min each at 40°C, 90% for 30min at room temperature, and 4 changes of 100% for 30min each at room temperature. The samples were infiltrated with propylene oxide (PO) 2 times for 30min each and put them into a 70:30 mixture of PO and resin (Quetol-812; Nisshin EM Co.,Tokyo, Japan) for 1h, then they kept the cap of tube open and PO was volatilized overnight. The samples were transferred to a resin (Quetol-812; Nisshin EM Co.,Tokyo, Japan), and polymerized at 600°C for 48h. The resin blocks were semi thin sectioned at 1.5 μm with glass knives using an ultramicrotome (ULTRACUT UCT;Lica) and stained with 0.5% Toluuidine blue.

The blocks were ultra-thin sectioned at 70 nm with a diamond knife using a ultramicrotome (ULTRACUT UCT;Lica) and sections were placed on copper grids. They were stained with 2% uranyl acetate at room temperature for 15min, and then rised with distillied water followed by being secondary-stained with Lead stain solution(Sigma-Aldrich Co.) at room temperature for 3 min. The grids were observed by a transmission electron microscope (JEM-1400Plus;JEOL Ltd.) at an acceleration voltage of 80 kV. Digital images (2048×2048 pixels) were taken with a CCD camera (VELETA; Olympus soft Imaging Solutions GmbH).

Western Blotting.

Briefly, brain tissues were immediately frozen in liquid nitrogen. The tissue and primary cells were lysed with lysis buffer (50 mM Tris-HCl, pH 7.5, 1 mM EDTA, 150 mM NaCl, 0.5% NP-40, 0.1% SDS and Complete protease inhibitor mixture (Roche Applied Science)) and homogenizing by sonication then subjected to immunoblotting as described elsewhere (Uchiumi et al., 2010). The lysates were clarified by centrifugation at 14,000 rpm for 10 min and quantified using Bio-Rad protein assay reagent. Signals were visualized with horseradish peroxidase (HRP)-conjugated anti-rabbit or anti-mouse IgG and with Super Signal West Dura substrate (Pierce). Chemiluminescence was recorded and quantified with a chilled charge-coupled device camera (LAS 4000) (Fuji film, Tokyo, Japan). Data shown are representative of three independent experiments. For quantification, we used the Multi Gauge software (Fuji film) and data acquired from the LAS 4000.

Preparation and Analysis of Neuron Culture in Vitro

Neuronal cell cultures were prepared from brain of P2 mice. Isolated brain were removed of meninges and cut into 0.5-mm^3 pieces. The cells were dissociated to single cells using a Nerve-Cell Dissociation media Kit (Sumitomo Bakelite). The resulting neuronal cells were suspended in pre-warmed primary neuron basal medium (PNBM, Lonza, Walkersville, MD) and primary neuronal growth medium (PNGM, SingleQuots Lonza) supplemented with NGF (100 ng/ml). Cells were plated at a density of 1000 cells/mm^2 on poly-l-lysine-coated plates for immunocytochemistry, or western blot analysis. Three days after plating, 50% of the medium was changed and subsequently the medium was changed every 3 days.

We isolate primary neuron, oligodendrocyte and astrocyte using the MACS® neural dissociation kit (Oligodendrocyte: Anti-O4 MicroBeads (#130-094-543), astrocyte: Anti-PSA-NCAM MicroBeads (#130-092-966), neuron: Neuron Isolation Kit (#130-098-752), respectively) (Miltenyi Biotec, Bergisch Gladbach, Germany). The Oligodendrocyte progenitor cell were cultures with Oligodendrocyte Precursor Cell Differentiation Medium (OPCDM) (Scien Cell). Astrocyte were cultured with Gibco Astrocyte Medium with N-2 Supplement X100, FBS certified one shot) (Gibco). The purity of the eluted O4-positive fraction was enriched about 90% by FACS analysis. For inhibition analysis, we added 1nM, 10nM Rotenone and 10mM 2-Deoxy Glucose at day 1 or at day 7 culture.

Neuron, astrocyte and Oligodendrocyte Culture

Neuron culture were performed with PNGM™ Medium (Lonza) which is conveniently

packaged in our BulletKit™ Format containing PNGM Primary Neuron Basal Medium (PNBM) (included Glucose and pyruvate) and the Primary Neuron Growth SingleQuots™ Kit (Lonza). The PNGM™ SingleQuots™ Kit contains the necessary volumes of 2mM L-glutamine, GA-1000, and 2% NSF-1, a supplement supporting neuronal growth and survival to complement the basal medium volume provided.

Oligodendrocyte were cultured with Oligodendrocyte Precursor Cell Differentiation Medium (OPCDM) (ScienceCell). OPCDM consists basal medium (5.55mM Glucose, 10mM Glutamine, 1mM pyruvate), 5 ml of fetal bovine serum (FBS), 5 ml of oligodendrocyte precursor cell differentiation supplement (OPCDS), and 5 ml of penicillin/streptomycin solution. It is a sterile, liquid medium which contains essential and non-essential amino acids, vitamins, organic and inorganic compounds, hormones, growth factors, trace minerals and a low concentration of fetal bovine serum (1%).

Astrocyte were cultures with GIBCO® Astrocyte Medium (Gibco) which has three components: base medium (DMEM), N-2 Supplement, and OneShot™ Fetal Bovine Serum (FBS). DMEM contained High Glucose, sodium pyruvate, GlutaMAX, phenol red. N-2 Supplement contained Human Transferrin, Insulin Recombinant Full Chain, Progesterone, Putrescine and Selenite. Mixing of these three components yields a complete medium.

Mitochondrial Complex activity O2 consumption assay

Mitochondrial activity assays were performed as described previously (Yagi et al., 2012). For mitochondrial isolation, nerves were dissected, desheathed, mechanically dissociated, digested with collagenase for 20 min and pooled together in 2 ml of homogenization buffer (HM) containing 0.22 M mannitol, 70 mM sucrose, 10 mM Tris-HCl, 0.5 mM EDTA, 1mM EGTA and 0.5% delipidated BSA. Nerves were homogenized for about 2 minutes (12 strokes) using a Teflon-glass homogenizer turning at 300 rpm. The homogenate was then centrifuged at 1000 x g for 10 min. The clarified supernatant was centrifuged at 15,000 x g for 10 min and the pellet was resuspended in 1ml of homogenization buffer and spun once more at 15,000 x g. The resulting pellet was resuspended in 250 µl of homogenization buffer without BSA. The protein concentration of the resuspended mitochondrial fractions was determined using a Coomassie (Bradford) Protein Assay Kit (Pierce). Five micrograms of protein from these mitochondrial preps were used to measure complex I-IV activity spectrophotometrically as previously described (Yagi et al., 2012).

Seahorse XF24 Flux Analyzer

The Seahorse XF24 Flux analyzer (Seahorse Biosciences, Billerica, MA) was used to determine the metabolic profiles of brain primary culture. 2 x 10^4 cells/well were seeded into Seahorse XF24 microplates and incubated at 37°C for approximately 4 days. Basal oxygen consumption rate (OCR) and extracellular acidification rate (ECAR) were measured in the Seahorse XF24 Flux analyzer. Additional measurements were performed after injection of four compounds affecting bioenergetics: (oligomycin (1 mM), carbonyl cyanide 4-trifluoromethoxyphenylhydrazone (FCCP) (300 nM), 2-deoxyglucose (2-DG), (100 mM), and rotenone (1 mM). Upon completion of the Seahorse XF24 Flux analysis, cells were trypsinized, counted, and the results were normalized per cells. Statistical analysis was performed using the t-test.

RNA Preparation and qRT-PCR

Total RNA was isolated after homogenization or lysis using a RNeasy Minikit (QIAGEN) according to the manufacturer’s protocol. RNA concentration was quantified using an ND-1000 spectrophotometer (Nanodrop Technologies). mRNA was reverse transcribed from 1 μg of total RNA using PrimeScript RT-PCR Kit (Takara). mRNA qRT-PCR was performed using a SYBR green-based detection system on a Step One Plus Sequence Detector instrument (Applied Biosystems) as described previously (Yagi et al., 2012). 18S ribosomal RNA expression was used to normalize samples and obtain relative expression values that were used to calculate fold changes.

mtDNA contents

For DNA isolation, tissue was digested and DNA isolated using DNeasy Blood and Tissue kit (QIAGEN) according to the manufacturer's protocol. DNA concentration was quantified using an ND-1000 spectrophotometer (Nanodrop Technologies). Mouse mitochondrial and nuclear copy number analysis was performed as previously described (Yagi et al., 2012). Briefly, genomic DNA preparations (also containing mitochondrial DNA) were quantitatively PCR’d in a Step One Plus Sequence Detector using ATP6 (mitochondrial) and AT3 (nuclear) primers. Calculations assumed 300,000 mitochondrial copies and 150 nuclear copies per ng input DNA. The primers used were: ATP6 forward, 5’- AGCTGGAGCCGTAATTACAG-3’ , reverse, 5’-TGTAAGCCGGACTGCTAATG-3’ and AT3 forward, 5’-AGTGGCAAATCGCGAATTGG-3’ , reverse, 5’-TGTGGACGACATCTGCATAG -3’

Free Fatty Acid Quantification

Brain FFA content was measured using the FFA quantification kit from BioVision following the manufacturer's instructions. 10mg tissue samples can be aetracted by homogenization with 200 μl of chloroform TritonX-100 (1% Triton X-100 in pure chloroform) in a microhomogenizer. Then spin the extract 5-10 minutes at top speed in a microcentrifuge. Collect organic phase (lower phase), air dry at 50°C to remove chloroform. Vacuum dry 30 min to remove trace chloroform. Dissolve the dried lipids (in Triton X-100) in 200 μl of Fatty Acid Assay Buffer by vortexing extensively for 5 min. Use 10 μl of the extracted sample per assay. Add 50 μl of the Reaction Mix and 2 μl Acyl-CoA Synthesis reagent to each well containing the samples. Incubate the reaction for 30 min at 37°C, protect from light. Fluorescence was measured using Corning black 96-well polypropylene assay plates and Measure O.D. 570 nm for colorimetric assay or fluorescence at Ex/Em = 535/590 nm in a micro-plate reader. FFA concentrations were calculated using a standard curve for palmitic acid ranging from 0 to 0.02 nmol/μL.

Image analysis

Quantification of Oligo2 positive cells and densitometric analysis of myelin, Tubulin and GFAP immunoreactivity. The number of Olig2-positive cells was determined in regions of white matter with an area of approximately 10 000 μm^2 using BZ-II Viewer software (Keyence). The colocalization of markers for CNPase, β-III Tubulin and GFAP was analyzed using BZ-X700 microscope (Keyence), and images were analyzed using BZ-II Viewer software (Keyence).

Carbamoyl Phosphate synthetase 1 (CPS1) activities

CPS1 activities assay was carried out as described by (Fahien and Cohen, 1964). The reaction was initiated by addition of the liver lysates to the rest of the reaction mixture. The reaction mixture contained 50mM Tris-HCl pH 8.0, 2.5 mM phosphoenopyruvate, 0.2 mM NADH, 30 mM NH_4Cl, 100 mM $KHCO_3$, 5 mM ATP, 10 mM $MgSO_4$, 10 mM N-acetylglutamate, 15 U/ml pyruvate kinase / lactate dehydrogenase (SIGMA P0294). The reactions were performed at 37°C and the decrease in absorbance at 340 nm was monitored. The initial velocity of the reaction was calculated to get the CPS1 activity. The activity assay was done with three pairs of p32 wild and cKO mice.

Ornithine transcarbamylase (OTC) activity

OTC activity in mouse brain was measured as described by Lee and Nussbaum (Lee and

Nussbaum, 1989) with modifications. Briefly, 2-10 μg of total cellular protein were added to 700μL of reaction mixture (5 mM ornithine, 15 mM carbamyl phosphate, and 270 mM triethanolamine, pH 7.7), which was incubated at 37 °C for 30 min. Reactions were stopped by adding 250 μL of 3:1 phosphoric acid/sulfuric acid (by volume). Citrulline production was then determined by adding 50 μL of 3% 2,3-butanedione monoxime, incubating at 95-100 °C in the dark for 15 min, and measuring absorbance at 490 nm.

Extraction of glycerophospholipids from mouse brain and LC-MS analysis.

We employed a modified Bligh and Dyer procedure. Briefly, after removed and frozen in liquid nitrogen, a lump of mouse brain (100-150 mg) was crushed with a small metal device by shaking using a MultiBeads Shocker (Yasui Kikai, Japan) at 2,000 rpm for 10 seconds. Phospholipids were then extracted with 1 ml of ice-cold 0.1N HCl-Methanol (1:1, v/v). After vortexing for 20 seconds, the samples were added with 500 μl of ice-cold chloroform and proceeded with vortexing for 20 seconds and centrifugation at 13,500 rpm for 1 min at 4°C. The lower organic phase was isolated and dried using a miVac DUO concentrator (GeneVac). The resulting lipid film was dissolved in 150 ul of methanol-chloroform (9:1, v/v) and added with 1 μl of ammonium hydroxide solution prior to LC-MS analysis.

The brain extracts were separated using high-performance LC (HPLC) on a Kinetex C8 column (150 × 2.1 mm, 1.7 μm particle size, Phenomenex, CA) coupled with a triple quadrupole mass spectrometer LCMS-8040 (Shimadzu, Japan). The mobile phase consisted of solvent A (10 mM ammonium formate) and solvent B (acetonitrile), and the column oven temperature was 53°C. The gradient elution program was as follows: a flow rate of 0.2 mL/min: 0–1 min, 2%B; 1-2 min, 2–67.5%B; 2–20 min, 67.5-92.5% B; 20-32 min, 92.5%B; 32–33 min, 92.5-100%B; 33-48 min, 100%B, 48-49 min, 100-2%B; and was maintained at 2%B until 55 min had passed. For ESI, the ionization parameters were as follows; drying gas flow rate, 10 L/min; nebulizer gas flow rate, 2 L/min; CDL temperature, 150 °C; DL temperature, 250ºC; and heat block temperature, 400°C. Detection of phosphatitylcholine (PC) and phosphatidylethanolamine (PE) was done in positive ionization mode by precursor ion scanning of a fragment ion of *m/z* 184 and by neutral loss scanning of 141 Da, respectively. On the other hand, phosphatidylserine (PS), phosphatidylinositol (PI), and phosphatidic acid (PA) were negative ionization mode by neutral loss scanning of 87 Da and precursor ion scanning of fragment ions of m/z 241 and 153, respectively. Other MS parameters including collision energy (CE) and scan *m/z*

scan range were as follows; PC (-20, 200-1000); PE (-25, 200-1000); PS (29, 200-1000); PI (45, 200-1000); PA (50, 200-1000).

LC-MS and data analysis.

The brain-derive metabolites were analyzed by LC-MS based on both reverse phase ion-pair chromatography and hydrophilic interaction chromatography (HILIC) modes coupled with a triple quadrupole mass spectrometer LCMS-8040 (Shimadzu). For monitoring metabolites including intermediates in central metabolism, a reverse phase ion-pair chromatography was performed using an ACQUITY UPLC BEH C18 column (100 × 2.1 mm, 1.7 µm particle size, Waters). The mobile phase consisted of solvent A (15 mM acetic acid and 10 mM tributylamine) and solvent B (methanol), and the column oven temperature was 40°C. The gradient elution program was as follows: a flow rate of 0.3 mL/min: 0–3 min, 0%B; 3-5 min, 0–40%B; 5–7 min, 40-100% B; 7-10 min, 100%B; 10.1–14 min, 0%B. Paremeters for negative ESI mode under multiple reaction monitoring (MRM) were as follows; drying gas flow rate, 15 L/min; nebulizer gas flow rate, 3 L/min; DL temperature, 250ºC; and heat block temperature, 400°C; collision energy (CE), 230kPa. On the other hand, for monitoring metabolites including amino acids, HILIC chromatography was performed using a Luna 3u HILIC 200A column (150 × 2 mm, 3 µm particle size, Phenomenex). The mobile phase consisted of solvent A (10mM ammonium formate in water) and solvent B(9:1 of acetonitrile:10 mM ammonium formate in water), and the column oven temperature was 40°C. The gradient elution program was as follows: a flow rate of 0.3 mL/min: 0–2.5 min, 100%B; 2.5-4 min, 100–50%B; 4–7.5 min, 50-5% B; 7.5-10 min, 5%B; 10.1–12.5 min, 100%B. Parameters for positive and negative ESI mode under MRM were as described above. Data processing was performed using LabSolutions LC-MS software program (Shimadzu, Japan) and statistical graphics were generate using the R statistical software program (http://cran.at.r-project.org/).

Antibody

The following primary antibodies were used Phospho-S6 Ribosomal Protein (Ser240/244) (#5364), Phospho-S6 Ribosomal Protein (Ser235/236) (#4858), S6 Ribosomal Protein (#2317), p-p70S6K (Thr389) (#9234), p70S6K(#2708), p-4EBP (Thr37/46) (#2855), p-4EBP (Ser65) (#9451), 4EBP (#9644), p-eIF4E(Ser209) (#9741), eIF4E (#2067), p-eIF2a (#3398), eIF2a (#9722), pAMPKa (Thr172)(#2535), AMPKa (#2603), p-AMPKb (#4181), AMPKb (#4178), p-AKT(Ser473) (#4060), p-AKT(Thr308) (#2965), AKT (#4691), p-mTOR(#2971), mTOR (#2983), p-Raptor

(#2083), Raptor (#2280), Phospho-ATP-Citrate Lyase (Ser455) (#4331), ATP-Citrate Lyase (#4332), caspase3 (#9662), Cleaved Caspase-3 (Asp175) (#9661), p-ACC (#3661), ACC (#3676), Neurofilament-L (#2837), Neurofilament-H (#2836), CNPase (#5664) GFAP (#3670) and purchased from Cell Signaling. Another primary antibodies were used OTC (ARP41766 avivasysbio.com), CPS1 (ab45956 abcam), Complex I NDUFA9(#459100 invitrogen), Complex II 70KDa Fp subunit (#459200 invitrogen), Ubiquinol-Cytochrome C Reductase Core Protein I (as110252 abcam), Complex V (alpha-subunit)(#439800 invitrogen), coxI(#459600 invitrogen), coxIII (#459300 invitrogen), olig2 (#9610 millipore), Myelin Basic Protein (ab40390 abcam), β-Tubulin III (T2200 SIGMA), respectively.

Secondary antibodies were used Anti-mouse IgG HRP-linked (#7076 cell signaling), Anti-rabbit IgG HRP-linked (#7074 cell signaling), Alexa Fluor® 488 F(ab')2 Fragment of Goat Anti-Rabbit IgG (H+L) (#A11070 Life Technologies), Alexa Fluor® 594 F(ab')2 Fragment of Goat Anti-Mouse IgG (H+L) (#A11020 Life Technologies).

REFERENCE

Fahien, L.A., and Cohen, P.P. (1964). A Kinetic Study of Carbamyl Phosphate Synthetase. J Biol Chem *239*, 1925-1934.

Lee, J.T., and Nussbaum, R.L. (1989). An arginine to glutamine mutation in residue 109 of human ornithine transcarbamylase completely abolishes enzymatic activity in Cos1 cells. J Clin Invest *84*, 1762-1766.

Uchiumi, T., Ohgaki, K., Yagi, M., Aoki, Y., Sakai, A., Matsumoto, S., and Kang, D. (2010). ERAL1 is associated with mitochondrial ribosome and elimination of ERAL1 leads to mitochondrial dysfunction and growth retardation. Nucleic Acids Res *38*, 5554-5568.

Yagi, M., Uchiumi, T., Takazaki, S., Okuno, B., Nomura, M., Yoshida, S., Kanki, T., and Kang, D. (2012). p32/gC1qR is indispensable for fetal development and mitochondrial translation: importance of its RNA-binding ability. Nucleic Acids Res *40*, 9717-9737.

Supplemental Table1 Metabolite changes in p32cKO white matter region (n=6)

metabolite	group	fold	*p value*
Serine	Amino acid	1.79	3.70E-06
Proline	Amino acid	1.78	2.04E-07
Phenylalanine	Amino acid	1.65	3.25E-03
Alanine	Amino acid	1.55	7.43E-06
Threonine	Amino acid	1.36	8.02E-04
Taurine	Amino acid	1.27	3.90E-07
Arginine	Amino acid	0.87	9.69E-03
Aspartic acid	Amino acid	0.77	5.62E-05
Glutamic acid	Amino acid	0.76	9.35E-07
gamma-Aminobutylic acid	Amino acid	0.70	1.73E-05
Butyrobetaine	Carnitine synthesis	1.59	1.65E-05
Acetylcarnitine	Carnitine synthesis	1.22	1.86E-03
Trimethyllysine	Carnitine synthesis	0.69	1.14E-10
Betaine	Carnitine synthesis	0.59	6.34E-07
Sarcosine	Creatine cycle	1.58	5.02E-07
Phosphocreatine	Creatine cycle	1.34	2.92E-03
Creatine	Creatine cycle	1.13	2.99E-03
Pyruvic acid	Glycolysis	3.15	1.59E-06
Fructose 6-phosphate	Glycolysis	1.24	7.84E-03
Lactic acid	Glycolysis	1.10	7.01E-04
Citric acid	Tricarboxylic acid cycle	1.99	1.29E-05
cis-Aconitic acid	Tricarboxylic acid cycle	1.78	6.85E-03
Ornitine	Urea cycle	2.06	7.34E-04
Citrulline	Urea cycle	0.85	5.59E-03
Putrescine	Others	7.51	4.49E-07
Orotic acid	Others	2.34	1.60E-05
2-Oxobutyric acid	Others	1.84	1.74E-03
Formylmethionine	Others	1.74	1.70E-04
3-Hydroxybutyric acid	Others	1.13	6.85E-04
Homocysteine	Others	0.81	2.14E-03
Glycerol 3-phosphate	Others	0.72	4.32E-09
Choline	Others	0.72	2.57E-05
Trimethylamine oxide	Others	0.43	1.97E-05
